# Janus Polymeric Nanorods Inhibit human Amylin Oligomerization and Fibrillation for Potential Type 2 Diabetes Treatment

**DOI:** 10.1002/smsc.70326

**Published:** 2026-07-27

**Authors:** Mathilde Jégo, Sandra Kalem, Irene Antignano, David Siefker, Mingsheng Ji, Julia Kaffy, Lynda Benrabah, Chloé Cayrou, Kawthar Bouchemal, Mélanie Hery, Erwan Nicol, Sandrine Pensec, Jutta Rieger, Claire Smadja, Sandrine Ongeri, Laurent Bouteiller, Olivier Colombani, Myriam Taverna

**Affiliations:** ^1^ Institut Galien Paris‐Saclay, UMR 8612 CNRS Université Paris‐Saclay Orsay France; ^2^ Institut des Molécules et Matériaux du Mans, UMR 6283 CNRS Le Mans Université Le Mans France; ^3^ Institut Parisien de Chimie Moléculaire, UMR 8232 CNRS Sorbonne Université Paris France; ^4^ BioCIS, UMR 8076 CNRS Université Paris‐Saclay Orsay France; ^5^ Chimie ParisTech Institut de Recherche de Chimie Paris, UMR 8247 CNRS PSL University Paris France

**Keywords:** fibrillation, *human islet amyloid polypeptide*, janus nanoparticles, oligomerization, type 2 diabetes

## Abstract

Protein misfolding and aggregation of human islet amyloid polypeptide (hIAPP) are central to β‐cell dysfunction in type 2 diabetes mellitus (T2DM). Despite many antiamyloid strategies, most suffer from poor selectivity, limited potency, and inadequate biocompatibility. Here, we report the first demonstration that polymeric Janus nanorods (JNRs) can delay both oligomerization and fibrillation of hIAPP through high‐affinity, sequence‐specific interactions. These anisotropic poly(N, N‐dimethylacrylamide) nanostructures, synthesized via reversible addition fragmentation chain transfer polymerization, self‐assemble to display a β‐hairpin mimetic ligand (LP2) on one face, enabling multivalent and directional binding. Biophysical assays reveal that JNR‐LP2 suppresses amyloid aggregation at substoichiometric concentrations, markedly outperforming free LP2 and nonfunctionalized JNRs. Mechanistic studies, including capillary zone electrophoresis and Thioflavin T fluorescence, show that JNR‐LP2 stabilizes monomeric hIAPP, prevents oligomer formation, and delays fibril nucleation. Importantly, binding experiments provide the first quantitative evidence of a strong affinity constant between a polymeric nanoparticle and hIAPP, establishing molecular specificity with negligible off‐target activity against unrelated amyloid proteins such as Tau. In cellular models, JNR‐LP2 mitigates hIAPP‐induced cytotoxicity while exhibiting excellent intrinsic biocompatibility. Collectively, these findings identify JNRs as a modular and selective nanoplatform for amyloid inhibition, offering a novel precision‐nanomedicine approach to T2DM.

## Graphical abstract

We demonstrate that polymeric Janus nanorods (JNRs) delay both oligomerization and fibrillation of human islet amyloid polypeptide (hIAPP) through high‐affinity and sequence‐specific interactions. JNRs display a β‐hairpin mimetic ligand (LP2) on one face, enabling multivalent binding. They stabilize monomeric hIAPP, prevent oligomer formation, and delay fibrillation. In cellular models, JNR‐LP2 mitigates hIAPP‐induced cytotoxicity and restores cell viability, while exhibiting excellent biocompatibility.

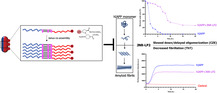

## 1 Introduction

Type 2 diabetes mellitus (T2DM) is a chronic, multifactorial metabolic disorder characterized by persistent hyperglycemia resulting from insulin resistance and the progressive failure of pancreatic β‐cells [1]. Affecting over 500 million individuals worldwide, T2DM poses a substantial public health burden and is closely linked to a range of comorbidities [2]. While traditionally associated with metabolic dysregulation, growing evidence highlights the critical role of protein misfolding and aggregation, particularly of human islet amyloid polypeptide (hIAPP), in the pathogenesis of T2DM [3]. hIAPP, or amylin, is a 37‐amino acid peptide co‐secreted with insulin by pancreatic β‐cells [4]. Under normal conditions, it regulates glucose homeostasis by inhibiting glucagon secretion, delaying gastric emptying, and promoting satiety [5]. In healthy β‐cells, interactions with insulin help stabilize hIAPP and prevent its aggregation [6, 7]. In T2DM, this protective mechanism is compromised, promoting pathological aggregation. hIAPP becomes prone to misfolding and aggregates via a nucleation‐dependent mechanism spanning monomers, oligomers, protofibrils, and mature fibrils [8, 9]. Soluble oligomers, in particular, are highly cytotoxic, disrupting membranes, inducing oxidative stress, triggering inflammatory cascades, and impairing mitochondrial function [10]. Amyloid plaques formed by hIAPP aggregates are observed in over 90% of individuals with T2DM and are strongly associated with β‐cell dysfunction and apoptosis [11]. Importantly, hIAPP aggregation occurs early in the disease course, potentially preceding clinical onset, underscoring its value as a therapeutic target [12].

Various classes of inhibitors have been developed to counteract hIAPP aggregation at different stages [13]. Small molecules, often featuring aromatic rings and polar functional groups such as Congo red, resveratrol, rifampicin, and morin, interfere with amyloid formation through π‐stacking and hydrogen bonding interactions [14]. Small peptides or peptide mimetics offer high biocompatibility and low immunogenicity and, through “like‐interacts‐with‐like” mechanisms, can disrupt hIAPP assembly [15, 16]. However, many of these inhibitors suffer from low specificity, limited potency, and suboptimal pharmacokinetics [17].

Therapeutic nanoparticles (NPs) have emerged as promising antiamyloid platforms, offering advantages over small molecules including multivalent binding, local enrichment of inhibitors, and tunable surface functionalization [17, 18]. Beside NPs used as carriers of encapsulated bioactive inhibitors, several nanomaterials, metal‐based NPs (gold, silver, and iron oxide) [19, 20, 21], carbon nanostructures (graphene quantum dots [GQDs] and carbon nanotubes) [22, 23, 24], and polymeric (dendrimers and micelles) or silica NPs [25, 26, 27, 28], have demonstrated their ability to modulate hIAPP aggregation through interaction with their surface. Yet, most of these systems primarily delay fibrillogenesis while leaving oligomeric intermediates largely unaddressed, despite their recognized cytotoxicity [29]. Recent approaches using inorganic platforms, such as iron oxide and gold NP–nanobody conjugates, have begun to target these early aggregates [19, 30], but issues of specificity, biocompatibility, and control over oligomerization remain unresolved. While some formulations partially disassemble mature fibrils, limited spatial control reduces their selectivity, and certain polymeric scaffolds can even accelerate fibrillation, as seen with poly(2‐hydroxyethyl acrylate) star polymers [27]. Collectively, these observations highlight the need for rationally designed nanoplatforms that integrate: (i) a ligand domain capable of selectively interfering with hIAPP aggregation and (ii) a particulate scaffold that enhances multivalent interactions.

**FIGURE 1:**
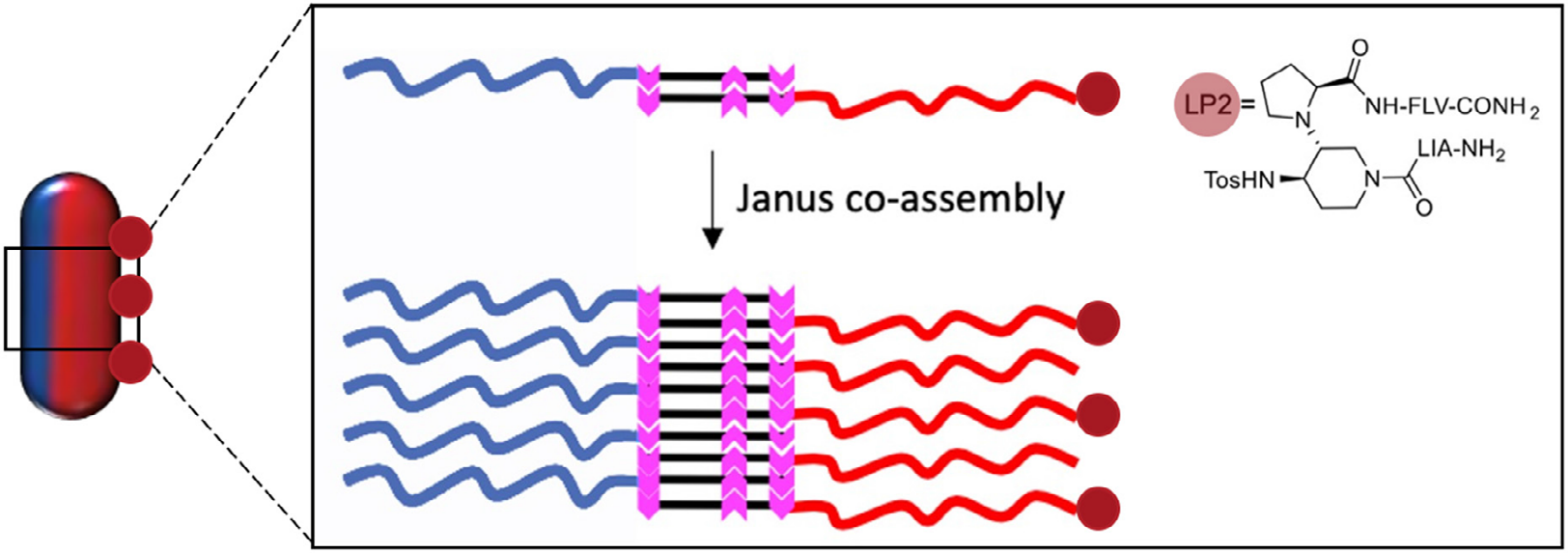
Schematic illustration of the JNRs used in this study. The blue and red arms on the scheme consist of polymer arms of the same chemical nature (PDMAc) and are distinguished in terms of color for the sake of clarity. The PDMAc arms on the blue side are fully nonfunctional, whereas some PDMAc arms on the red side are functionalized with LP2 ligands (formula shown in the insert). The final panel depicts the resulting nanorod architecture incorporating the ligand‐functionalized polymers.

Here, we introduce a novel class of anisotropic polymeric NPs, Janus nanorods (JNRs), engineered to selectively inhibit both hIAPP oligomerization and fibrillation. These nanorods are formed via H‐bonding‐driven supramolecular assembly of poly(*N*, *N*‐dimethylacrylamide) (PDMAc)‐based precursors bearing asymmetric trisurea stickers [31], yielding a distinct Janus architecture (Figure 1). Their small diameter, in the 10 nm range [32], should grant then a high specific surface allowing efficient hIAPP capture, while their high length, in the hundreds of nm range, should prevent their internalization in cells [33, 34] so that they do not become toxic.

Our long‐term objective is to rely on the possibility of functionalizing one face of these Janus particles with ligands able to inhibit hIAPP aggregation and the other face with functionalities: i) directing the JNRs to specific parts of the body, like pancreatic cells, to enhance their efficiency or ii) delivering a second bioactive molecule effective in T2DM creating multitarget NPs. In this article, only one side of the JNRs was functionalized in order to prove the concept that decorating these anisotropic and nanometric particles with hIAPP aggregation inhibitors would result in improved efficiency compared to free ligands without causing cell toxicity, thereby increasing the viability of pancreatic cells in the presence of hIAPP. One face of the JNRs is therefore unmodified to allow passive interactions with plasma proteins to eventually prolong systemic circulation. The opposite face is functionalized with a β‐hairpin mimetic ligand (LP2) integrating a piperidine–pyrrolidine β‐turn inducer and two sequence‐recognition elements (A25IL27 and F15LV17). LP2 was previously shown in monomeric form to delay both hIAPP oligomerization and fibrillation [35] but only at high micromolar concentrations (∼100 µM). By presenting LP2 multivalently on JNRs, we achieve potent inhibition at substoichiometric levels, exploiting both increased local ligand concentration and anisotropic, directional presentation. Multivalent interactions are thermodynamically favored and allow stronger and more selective interference with aggregation pathways compared to monovalent ligands [36, 37]. Crucially, we demonstrate for the first time that polymeric JNRs bind hIAPP with high affinity, disrupt oligomerisation, and delay fibril nucleation. Together, these properties establish functionalized polymeric JNRs as a modular, biocompatible strategy for precision modulation of amyloid aggregation in T2DM.

This study details the design, synthesis, and functionalization of anisotropic JNRs bearing LP2 ligands. Thioflavin T (ThT) fluorescence assays monitored fibril formation kinetics [38], while capillary zone electrophoresis (CZE) enabled quantitative, real‐time monitoring of soluble hIAPP oligomers at near‐physiological concentrations [39]. To directly determine binding affinities under native‐like conditions, we applied a label‐free frontal analysis continuous capillary electrophoresis (FACCE) approach [40]. Finally, cell viability studies assessed the protective effects of JNRs against hIAPP‐induced cytotoxicity. Together, these experiments provide a quantitative evaluation of JNR efficacy, supporting their potential as a therapeutic strategy for modulating hIAPP toxicity in T2DM.

## 2 Results and Discussion

### 2.1 Design and Characterization of nJNR and JNR‐LP2

#### 2.1.1 Ligand and Polymer Synthesis

**SCHEME 1:**
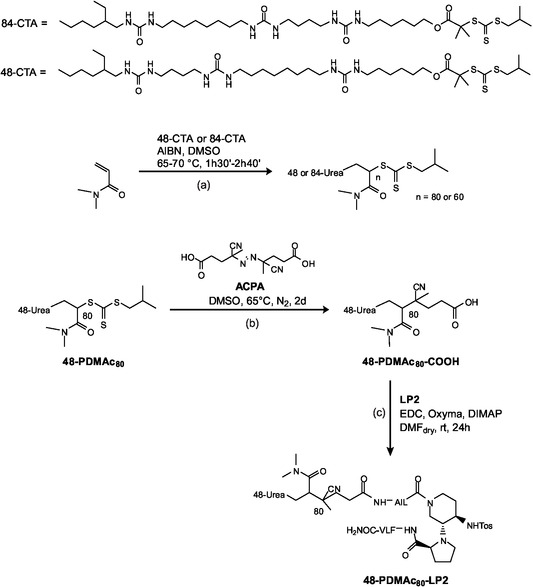
On the top: chemical structure of 48‐CTA and 84‐CTA. (a) Synthesis of 48‐PDMAc_80_ and 84‐PDMAc_60_ by RAFT polymerization. (b) Transformation of the trithiocarbonate end group of 48‐PDMAc_80_ into a carboxylic acid via radical coupling. (c) Coupling of 48‐PDMAc_80_‐COOH with LP2 ligand via amidation reaction.

The polymers 48‐PDMAc_80_ and 84‐PDMAc_60_ (Scheme 1a) were synthesized by reversible addition fragmentation chain transfer (RAFT) polymerization using two trisurea‐functional chain transfer agents (CTAs), namely 48‐CTA or 84‐CTA (Scheme 1, top). The synthesis of the CTA stickers has been previously reported [31], and the conditions for the RAFT polymerization are described in Section 3, with the characterization of the polymers reported in the Supporting Information (Figures S1–S3).

The LP2 ligand was synthesized following a previously described procedure [35]. Briefly, three amino acids, leucine (L), isoleucine (I), and alanine (A), were sequentially coupled to the N‐terminus of the piperidine–pyrrolidine scaffold. Subsequently, the tripeptide FLV‐NH_2_ was conjugated to the carboxyl group of the pyrrolidine ring. In the final step, acidic cleavage of the Boc‐protecting group on the terminal alanine yielded LP2 as a free amine.

In order to functionalize the 48‐PDMAc_80_ with the peptide LP2, it was necessary to transform the trithiocarbonate end group into a carboxylic acid, which was achieved by radical coupling using an excess of 4,4′‐azobis(4‐cyanopentanoic acid (ACPA) according to a procedure previously described [41] (Scheme 1b). The removal of the trithiocarbonate group was verified by recording the UV‐vis spectrum of the polymer before and after the reaction (Figure S4). The disappearance of the characteristic band with *λ*_max_ = 309 nm related to the trithiocarbonate group was observed, indicating a complete transformation of the polymer end group. The resulting product was also characterized by size exclusion chromatography (SEC) and ^1^H‐nuclear magnetic resonance (NMR) (Figures S5 and S6). The acid‐terminated polymer 48‐PDMAc_80_‐COOH was then coupled with the LP2 ligand through a carbodiimide‐activated amidation process. Due to the bulky nature of the LP2 ligand, the use of 1‐ethyl‐3‐(3‐dimethylaminopropyl)carbodiimide (EDC) as a coupling agent, Oxyma as an additive, and DIMAP as a base was required to promote the coupling of the LP2 ligand to the acid‐terminated polymer (Scheme 1c). Comparison of the refractive index (RI) signal obtained by SEC of the purified 48‐PDMAc_80_‐LP2 and LP2 ligand alone (Figure S7) shows that there is no free peptide in the obtained polymer. ^1^H‐NMR confirms the presence of the ligand, which is, according to SEC, covalently coupled to the polymer. Furthermore, the ^1^H‐NMR analysis shows that 30 mol% of the polymer is functionalized with the LP2 ligand (relative integration of the reference peak at 0.2–1.0 ppm and the aromatic protons of LP2 at 7.0–8.0 ppm, Figure S8).

#### 2.1.2 JNR CoAssembly and Physicochemical Characterization

A “DMSO/water” protocol, wherein the polymers were initially solubilized in dimethyl sulfoxide (DMSO) and subsequently mixed with a large excess of water, was employed to promote coassembly into elongated nanocylinders. This method, previously established for a different polymer pair, was adopted here under analogous reference conditions [31, 32]. The resulting supramolecular JNRs formed in the aqueous medium were kinetically trapped and thus remained in a nonequilibrium state.

##### 2.1.2.1 Determination of the JNR Size and Morphology

**FIGURE 2:**
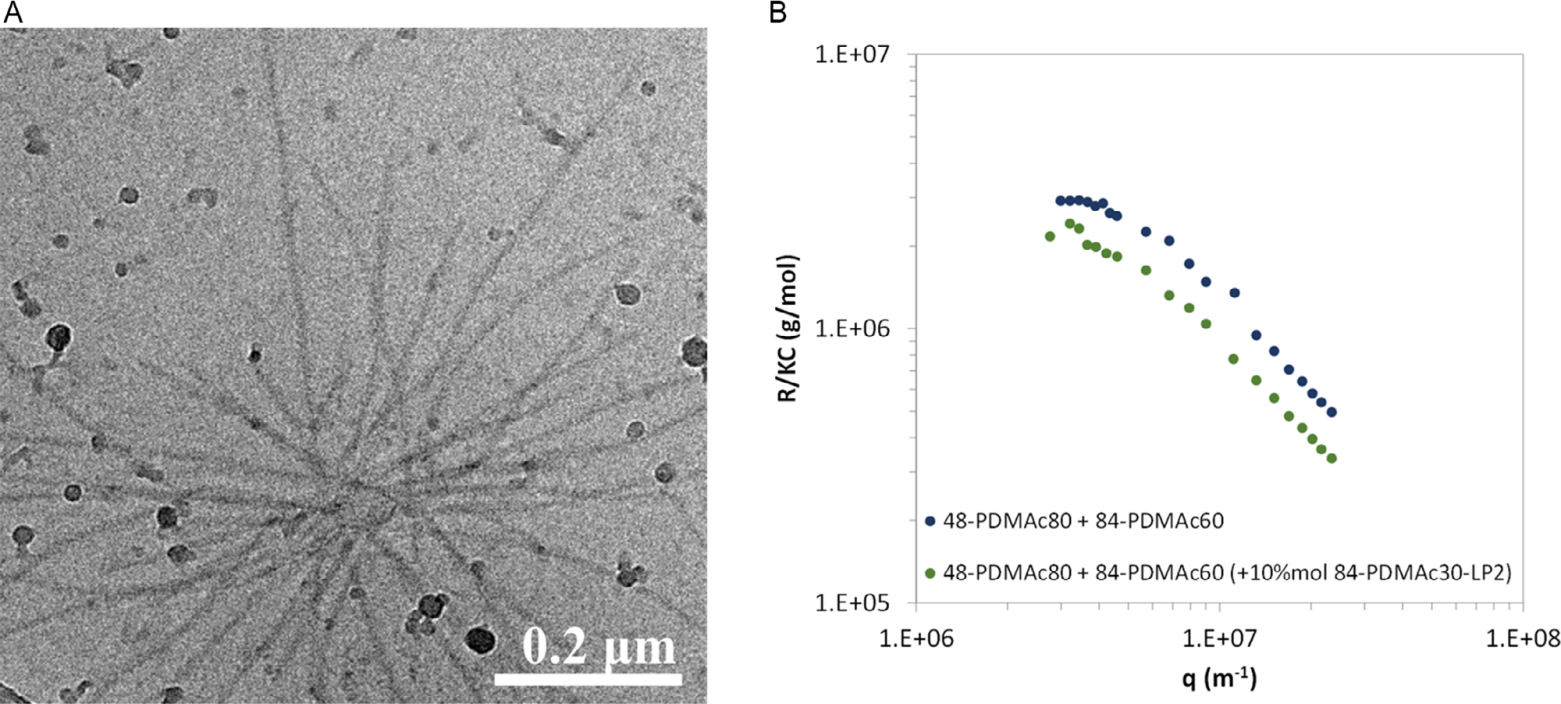
Reference conditions to form nanocylinders: (48‐PDMAc_80_ + 84‐PDMAc_60_) equimolar mixture (0.9 g/L in DMSO/water (1/99 v/v) obtained starting from *C*_0_ = 90 g/L in DMSO, with an addition flow rate of water *R* = 0.5 mL/h at *T* = 20°C until a final polymer concentration *C*_f_ = 0.9 g/L. The solution was dialyzed against water 24 h to remove DMSO. (A) Cryo‐TEM image of the nJNR nanocylinders after dialysis and (B) SLS data for nJNR (•) and JNR‐LP2 (•).

The first system, consisting of nJNR, was investigated. Cryogenic‐transmission electron microscopy (cryo‐TEM) revealed rod‐shaped structures that measure several hundred nanometers in length and exhibit a rather uniform diameter of approximately 10 nm (Figure 2A). Static light scattering (SLS) data show that the scattered intensity displays a q^−1^ dependency for 3.10^6^ m^−1^ < *q* < 10^8^ m^−1^, which confirms the formation of rigid cylindrical structures (Figure 2A). In the q‐range where the scattered intensity I scales as q^−1^, the molecular weight per nanometer along the main axis of the supramolecular nanocylinders, *M*_L_, can be determined: *M*_L_ = 4.8 × 10^3^ g.mol^−1^.nm^−1^. Considering that *M*_unimer_ of the polymer = 8.5 × 10^3^ g/mol (corresponding to the average *M*_w_ of an equimolar mixture of 48‐PDMAc_80_ and 84‐PDMAc_60_), that the distance between two hydrogen‐bonded urea units is *e* = 0.46 nm, and making the reasonable assumption that the hydrogen bonds are formed in the direction of the cylinder axis, it follows that the cross‐section of the nanocylinders contains *M*_L_/*M*_unimer_ = 0.3 molecules. Assuming that the light intensity scattered by unimers is negligible compared to that scattered by JNR, this implies that 30% of the polymer self‐assembled into JNR; the rest remaining as unimers [32]. At the minimum *q* investigated by SLS, the weight average molar mass of the nanocylinders reached a plateau (*M*_w_ = 3 × 10^6^ g/mol), allowing the calculation of the aggregation number of the nanocylinders, taking into account that only 30% of the polymers self‐assembled (*N*_agg_ = *M*_w_/(30%.*M*_w,unimer_) = 1050 with *M*_w,unimer_ = 8.5 × 10^3^ g/mol) and therefore a length *L* = *N*_agg_ × 0.46 nm = 480 nm. Dynamic light scattering (DLS) measurements, performed on filtered samples, revealed the existence of only one population of scatterers with an average hydrodynamic radius *R*_h_ = 70 nm. Given that the particles are not spheres but nanocylinders, the hydrodynamic radius does not correspond to their size but to the size of a sphere with the same diffusion coefficient. Using *R*_h_ and the diameter of the nanocylinders estimated by cryo‐TEM, the length of the nanocylinders was calculated to be approximately 600 nm following a previously reported approach [32]. The lengths of the JNRs determined by SLS and DLS are in good agreement. The small difference can be attributed to the fact that SLS leads to a weight average molar mass (and length), whereas DLS affords a z‐average length, the latter being influenced by the longest particles. Both values are also in good agreement with the dimensions observed by cryo‐TEM (Figure 2).

For the JNR‐LP2, 10% molar of 48‐PDMAc_80_‐LP2 was attached to the 48‐PDMAc side (out of which 30 mol% are effectively functional, see Section 2.1.1). In this case, SLS results also showed a q^−1^ dependency, which confirms the formation of rigid cylindrical structures (Figure 2A). The percentage of nanocylinders was also calculated, and 20% of nanocylinders was found. This indicates that approximately 80% of the polymers failed to assemble. Although this factor must be considered in the analysis, the magnitude of the unassembled fraction remains comparable with or without LP2, as well as their average length and aggregation number. Considering that JNR‐LP2 consists of about 1000 polymer arms (*N*_agg_), 500 on each side, and that the 48‐PDMAc side bears ∼3 mol% of LP2 functions (10 mol% of 48‐PDMAc_80_‐LP2 among the 48‐PDMAc chains, out of which only 30 mol% are effectively functionalized, see part 2.1.1), this implies that each JNR‐LP2 contains about 15 LP2 functions. This value should be considered as an average number of LP2 per JNR since JNRs are actually polydisperse in length.

Overall, cryo‐TEM, SLS, and DLS gave consistent information and confirmed the formation with these process conditions of long (micron‐range), thin (*d* = 10 nm), and rigid nanorods.

##### 2.1.2.2 Zeta Potential Measurements

Zeta potential measurements were performed on nJNRs dispersed in 100 mM bicine buffer (pH 7.4), a zwitterionic medium offering sufficient ionic conductivity (∼502 µS/cm) for reliable electrophoretic analysis without added salts [42]. Despite being composed of nonionizable, neutral polymer arms, nJNRs exhibited a consistent, very low negative zeta potential (–13.6 ± 1.0 mV). While this value does not suggest strong electrostatic stabilization, it suggests a measurable electrophoretic mobility under conditions relevant for FACCE experiments performed later in this study.

Long‐term measurements confirmed stability over 6 months at 4°C, with only minor variation (–12.9 ± 0.8 mV at 10 days, –12.5 ± 0.4 mV at 1 month, and –11.2 ± 0.9 mV at 6 months), indicating preserved surface properties and colloidal integrity.

### 2.2 Effect of the JNR on hIAPP Aggregation

To comprehensively evaluate the impact of the JNR on the aggregation of hIAPP, we employed two complementary in vitro techniques: ThT fluorescence assays and CZE. These methods provide distinct yet synergistic insights into the aggregation process. ThT assays are highly sensitive to the formation of cross‐β‐sheet‐rich fibrillar structures, allowing quantitative monitoring of the late stages of amyloid fibril formation [38]. However, ThT does not detect early soluble oligomers or transient intermediates that preceded fibril growth. Conversely, CZE offers high‐resolution, real‐time detection of soluble species, including monomers, oligomers composed of a limited number of monomers, and transient intermediates that are typically invisible to ThT. This double approach enables us to capture both the early molecular events via CZE and the fibrillation endpoint dynamics via ThT, providing a comprehensive understanding of how the JNR modulates not only the final aggregation outcome but also the underlying oligomerization pathways.

In order to rationalize the results, CZE and ThT experiments were conducted with JNR‐LP2, functionalized with 3 mol% of LP2 functions on the 48‐PDMAc_80_ side, and compared to nonfunctionalized JNR (nJNR) as well as with free LP2. JNR‐LP2 and free LP2 assays were conducted with the same total molar concentration of LP2 ligands to allow relevant comparison (see Section 3.5.2). Since nJNRs bear no LP2, they were prepared at the same weight concentration (g/L) as JNR‐LP2 to allow distinguishing the role of the backbone of the JNR from that of the ligands attached to JNR‐LP2 during the experiments.

#### 2.2.1 Inhibition of hIAPP Oligomerization by CZE

**FIGURE 3:**
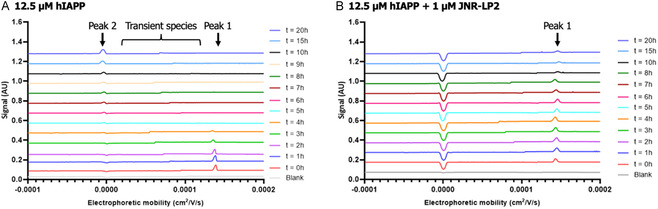
Real‐time CZE monitoring of hIAPP oligomerization at 12.5 µM over 20 h in the absence (A) and presence (B) of 1 µM JNR‐LP2 (the molar concentration indicated here corresponds to that of LP2 ligand, not of JNRs). Separations were performed using a polybrene‐coated fused silica capillary (60 cm total length, 50 cm to detector) to minimize peptide adsorption. hIAPP samples were hydrodynamically injected from the long end of the capillary (20 psi) and separated under −25 kV at 25°C in 50 mM ammonium acetate buffer (pH 3.7). UV detection was carried out at 200 nm. Peaks correspond to monomeric hIAPP (peak 1), transient oligomeric intermediates (broad signals between peaks 1 and 2), and higher‐order soluble oligomers comigrating with the EOF (Peak 2).

CZE was employed to monitor, in real‐time, the oligomerization process of hIAPP in solution, both in the presence and absence of potential inhibitors. Three forms of inhibitors were tested: the free LP2 molecule, nJNR, and functionalized JNR (JNR‐LP2). The CZE method used in this study was originally developed and validated by our group for hIAPP at 100 µM [39]. In the present study, a lower peptide concentration of 12.5 µM was chosen to better reflect biologically relevant conditions. This adjustment is primarily justified by the need to avoid excessive cytotoxicity, as hIAPP concentrations near 100 µM are known to induce severe toxicity in β‐cell models [43]. Additionally, the use of lower concentrations mitigates the rapid aggregation kinetics observed at higher ones, enabling more accurate monitoring of early molecular interactions between hIAPP and NPs. From an analytical perspective, the method demonstrates high sensitivity at a detection wavelength of 200 nm, allowing for reliable detection at 12.5 µM of hIAPP without compromising resolution. Importantly, the electropherograms obtained for hIAPP without inhibitor at 12.5 µM (Figure 3A) exhibited comparable separation profiles to those previously observed at 100 µM (Figure S9).

To better mimic physiological conditions, attempts were made to perform the kinetic analysis at pH 7.4 instead of pH 3.7. However, these were unsuccessful due to the markedly accelerated aggregation kinetics, which prevented the temporal resolution of intermediate species. Therefore, peak assignments were established at pH 3.7, in the absence of inhibitors, based on our previous work [39]. Peak 1 corresponds predominantly to monomeric hIAPP, though minor contributions from dimers or trimers cannot be entirely ruled out. For clarity, this peak is referred to as monomeric hIAPP throughout the article. Peak 2 represents higher‐order soluble oligomers that comigrate with the electroosmotic flow (EOF). Between these two primary peaks, additional signals were observed, corresponding to transient oligomeric intermediates with variable electrophoretic mobilities. These likely reflect small, dynamically exchanging species composed of a limited number of monomers (typically tetramers to hexamers). Their fluctuating hydrodynamic radii and charge‐to‐mass ratios during the aggregation process give rise to broad and diffuse migration profiles in the electropherogram [44].

Importantly, the method demonstrated excellent precision and reproducibility. Across three independent hIAPP batches analyzed over 3 months, the relative standard deviation (RSD) for peak migration times was 1.5%, and the maximum RSD for peak areas was 1.4% (Figure S10A). Intrabatch reproducibility over four consecutive days showed RSDs of 1.9% for electrophoretic mobility and 1.4% for peak area (Figure S10B), confirming the reproducibility of the CZE method for quantitative kinetic studies at physiologically relevant concentrations.

**FIGURE 4:**
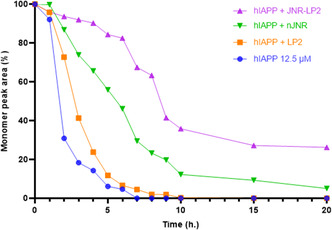
Kinetics of monomeric hIAPP disappearance in the presence of JNR‐LP2, nJNR, and free LP2. The concentration of monomeric hIAPP (12.5 µM) was monitored over time either alone (control) or with JNR‐LP2 and free LP2, both tested at a substoichiometric molar ratio of 12.5:1 (hIAPP: LP2 ligand). For nJNR, which lacks LP2 ligands and cannot be compared on a molar basis, the same mass concentration as that used for JNR‐LP2 was applied.

To evaluate the inhibitory efficacy of the different compounds, 12.5 µM hIAPP was incubated with JNR‐LP2, nJNR, or free LP2. To investigate the impact of ligand presentation, free LP2 and JNR‐LP2 were tested under the same molar ratio of hIAPP peptides to LP2 functions (12.5:1), either free or bound on JNR‐LP2. In addition, the weight concentration of nJNR used as a control in this experiment was kept identical to the weight concentration of JNR‐LP2. Figure 4 depicts the percentage of hIAPP monomer remaining over time, which indicates the tested compounds’ capacity to preserve monomeric hIAPP during the early aggregation stages. JNR‐LP2 demonstrated a marked alteration in the aggregation kinetics of hIAPP compared to free LP2, significantly extending the lifetime of soluble monomeric hIAPP. Remarkably, even at a substoichiometric molar ratio of hIAPP:JNR‐LP2 (12.5:1), JNR‐LP2 significantly prolonged the stability of monomeric hIAPP, with over 35% remaining after 10 h. Under these conditions, the oligomeric peak (peak 2, attributed to higher‐order oligomers) does not appear (Figure 3B), indicating effective stabilization of hIAPP in its monomeric form and prevention of higher‐order aggregate formation.

Interestingly, control experiments with nJNR showed that even the unfunctionalized nanorod suppressed the oligomeric peak (Figure S11B), indicating that nonspecific interactions with the scaffold can contribute to aggregation inhibition. However, this effect was considerably weaker than that of JNR‐LP2: in the presence of nJNR, monomer levels fell below 12% within just 10 h (Figure 4, green curve), highlighting the essential role of LP2 functionalization in sustaining hIAPP monomer stability over time. The advantage of ligand attachment became even more apparent when comparing JNR‐LP2 to free LP2 at the same molar ratio (12.5:1). Free LP2 produced only a slight delay in oligomerization, with monomeric hIAPP dropping to 10% within 5 h, whereas more than 80% of hIAPP remained monomeric in the presence of JNR‐LP2 at an identical molar concentration of LP2 ligands (Figure 4, orange vs. purple curves). Moreover, free LP2 failed to abolish the oligomeric signal entirely, as peak 2 persisted throughout the experiment (Figure S11). Thus, the difference in inhibition efficiency between JNR‐LP2 and free LP2 far exceeds that between free LP2 and the untreated control (hIAPP alone) underscoring the superior impact of the multivalent, nanostructured presentation.

Taken together, these results demonstrate that the strong inhibitory effect of JNR‐LP2 is not solely due to the unspecific action of nJNR combined with the inhibitory role of LP2 ligands but results from a synergistic effect, which we attribute to the multiple functionalities of the JNR‐LP2 inhibitor. Each JNR‐LP2 nanocylinders indeed bear about 15 LP2 ligands (see Section 2.1.2.1), which probably enhances their ability to capture hIAPP and hIAPP oligomers.

**FIGURE 5:**
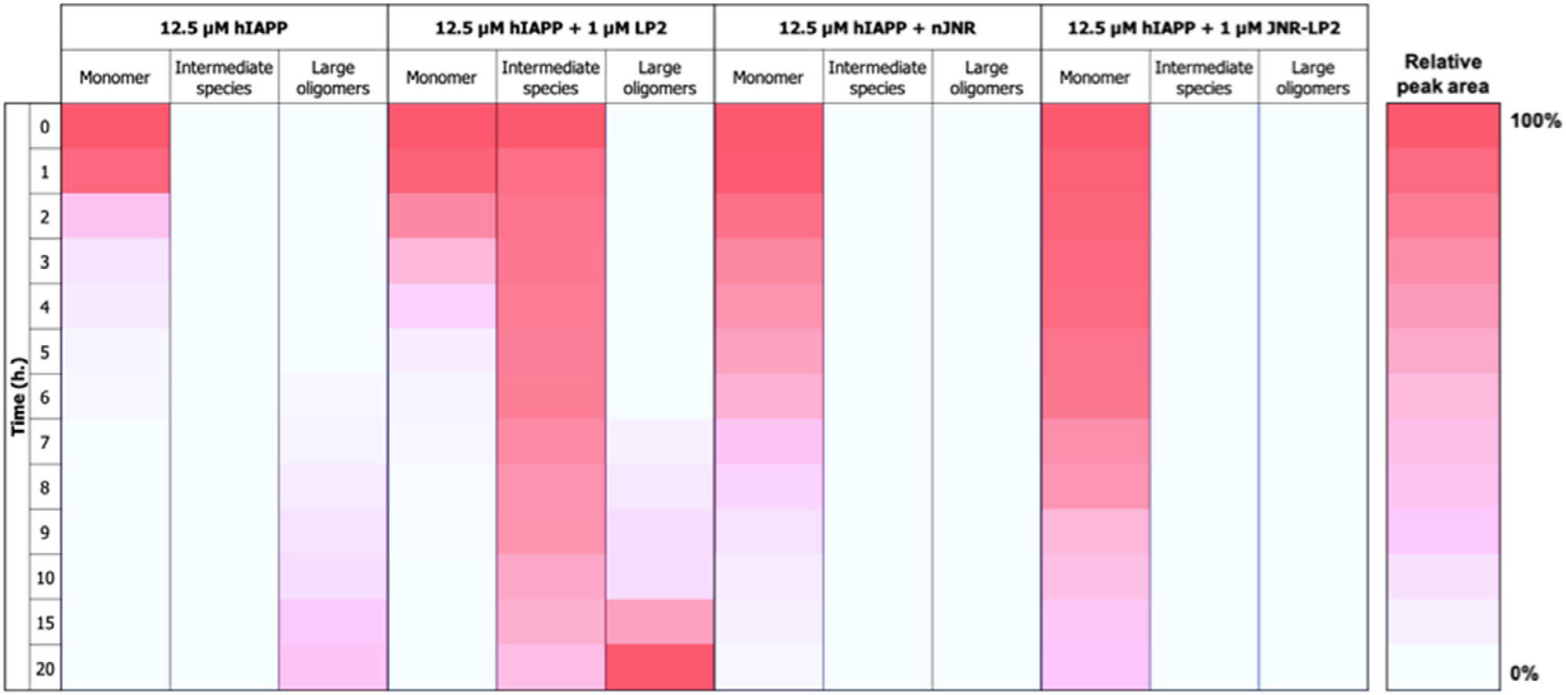
Heatmap visualization of the oligomerization dynamics of hIAPP at 12.5 µM in the absence and presence of nJNR, JNR‐LP2, and LP2, as monitored by CZE. Rows represent incubation time, and columns represent the different conditions (hIAPP control, LP2, nJNR, and JNR‐LP2). Color intensity reflects the relative abundance (relative peak area in percentage) of transient oligomeric intermediates and larger oligomers (red = higher abundance and white = lower abundance). The peak area of larger oligomers was estimated considering that the EOF peak did not produce a significant signal.

To further dissect the aggregation mechanism of hIAPP and the impact of our different compounds, we generated a heatmap that globally visualizes the temporal evolution of the proportion (relative peak area) of all detectable species, including transient intermediates (Figure 5). Unlike the kinetic profiles in Figure 4, which primarily focus on the monomer, this mapping provides detailed insight into the dynamic presence and modulation of intermediate species along the aggregation pathway. In the control experiment (hIAPP alone), the heatmap reveals a transient yet pronounced presence of intermediate species during the early phases of aggregation (between 1 and 8 h), which then progressively shift toward higher‐order oligomers. This suggests a classical nucleation‐dependent polymerization mechanism where transient oligomers serve as intermediates toward fibril formation [45].

Interestingly, while the inhibitory effect of free LP2 has been previously reported [35], our findings reveal for the first time that even at very low concentrations, and notably at substoichiometric ratios, LP2 can significantly reduce oligomer formation and promote the persistence of monomeric hIAPP species. Despite this, free LP2 alone does not entirely prevent the formation of transient oligomeric intermediates or even stabilize them. Notably, the elevated levels of these intermediates cannot be directly attributed to LP2 itself, as the LP2‐associated peak on the electropherogram does not overlap with any hIAPP‐related species (Figure S12). This suggests that LP2 may form self‐aggregates or noncovalent complexes, primarily with monomeric hIAPP, without significantly interfering with oligomer formation, or at least through a slight retardation. Consequently, a portion of unbound or insufficiently inhibited hIAPP monomers likely continues to transition into oligomeric forms, highlighting the limited inhibitory efficacy of free LP2 in isolation.

In contrast, under both nJNR and JNR‐LP2 conditions, transient oligomeric species are completely suppressed throughout the 20‐h incubation period (Figure 5, columns 3–4). This observation indicates that the nanostructured systems do not merely slow the aggregation process but fundamentally alter the oligomerization pathway. This likely occurs through effective sequestration of monomeric hIAPP, thereby preventing the nucleation and growth of aggregation‐prone intermediates.

Furthermore, a superior inhibitory effect is observed in the JNR‐LP2 condition compared to nJNR alone. This is evidenced by a more pronounced and sustained suppression of oligomeric hIAPP, suggesting enhanced binding affinity or structural compatibility between LP2‐functionalized nanostructures and the peptide. These findings imply that, beyond simply inhibiting fibril elongation, JNR‐LP2 profoundly impacts the aggregation mechanism by eliminating key transient species that typically serve as nucleation seeds. Importantly, this mechanistic insight extends beyond what could be inferred from CZE kinetic traces alone. It emerges more clearly through the integrative analysis enabled by the heatmap, offering a comprehensive view of how the aggregation pathway behind the different inhibitor systems can vary.

#### 2.2.2 Inhibition of hIAPP Fibrillization by ThT Fluorescence Assay

ThT is a fluorescent dye that specifically binds to cross‐β sheet‐rich structures characteristic of amyloid fibrils [46]. Upon binding, its fluorescence increases, making it a gold‐standard tool for monitoring the aggregation kinetics of amyloidogenic proteins such as hIAPP [47]. Notably, ThT does not interact with small oligomeric species of hIAPP and thus cannot capture early aggregation events. Its use, therefore, provides a complementary perspective to CZE, allowing us to specifically track the progression toward fibril formation.

**FIGURE 6:**
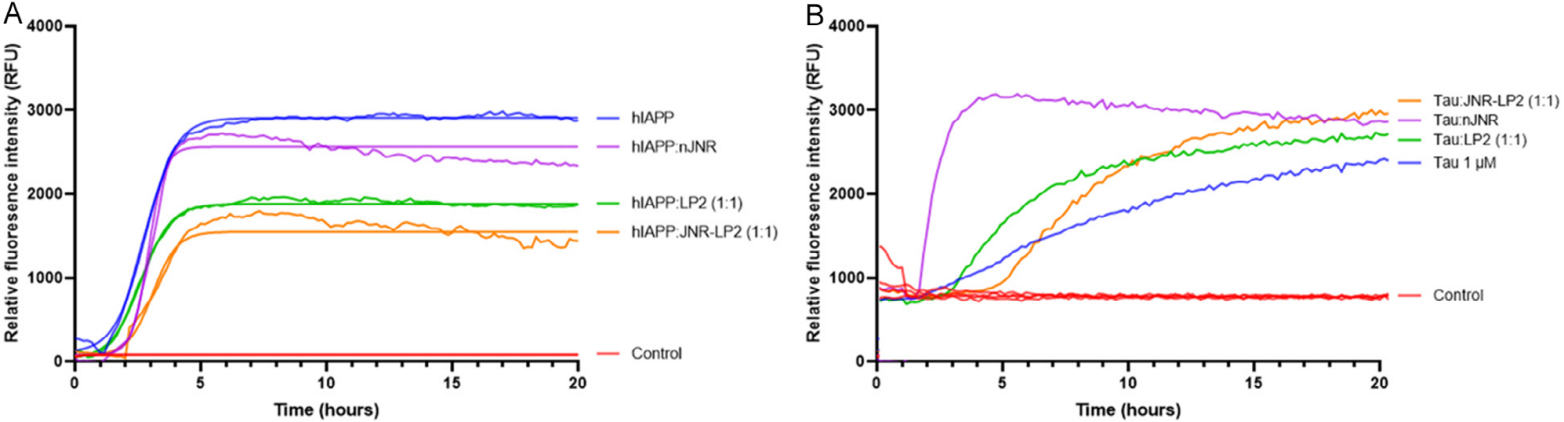
ThT fluorescence monitoring of amyloid fibril formation for hIAPP and Tau. Aggregation kinetics of hIAPP (A) and Tau (B) at 1 µM were monitored over time. For hIAPP, the curves were fitted to a Boltzmann sigmoidal model. The blue curves represent the aggregation of each peptide in the absence of any compound. The effects of LP2 (hIAPP:LP2 ligands 1:1 molar ratio, green), JNR‐LP2 (hIAPP:LP2 ligands 1:1 molar ratio, orange), and nJNR (same weight concentration as for JNR‐LP2, purple) are shown for comparison. Control samples containing only buffer are shown in red. Each hIAPP curve represents the mean of three independent experiments, with standard deviations reported in Table 1. For Tau, a representative curve from one of three replicates is shown (no fitting applied).

The aggregation profile of hIAPP alone exhibits the typical sigmoidal curve (Figure 6, blue curve), composed of (i) a lag phase (∼1 h), corresponding to the nucleation period during which monomers and transient low‐n oligomers (dimers to hexamers) progressively assemble but do not yet form β‐sheet‐rich nuclei detectable by ThT, (ii) an elongation phase (∼1–5 h), marked by a sharp increase in fluorescence, which reflects the rapid conversion of nucleation seeds into β‐sheet‐rich fibrillar structures, and (iii) a plateau phase (after ∼5 h), where the reaction reaches equilibrium [48]. Importantly, the height of the plateau does not directly reflect only the total mass of fibrils but is also influenced by fibril structural features, including the degree of β‐sheet stacking, fibril length, morphology, and the number of accessible ThT‐binding sites [49]. Two key parameters were extracted to assess the effect of inhibitors on aggregation: the maximum fluorescence intensity at the plateau (F), which provides an indirect measure of fibril load and/or fibril structural properties, and the half‐time of aggregation (*t*_1__/__2_), corresponding to the time required to reach 50% of the maximal fluorescence, reflecting the aggregation kinetics.

ThT fluorescence measurements revealed that the height of the plateau on Figure 6A followed the order hIAPP without inhibitor > nJNR > free LP2 > JNR‐LP2. This result associated with a more quantitative analysis (Table 1) implies that nJNR had an effect, but a very modest one, on fibril yield, reducing the final fluorescence signal by ∼10%. Free LP2 had a more pronounced effect than nJNR. Regarding *t*_1/2_, nJNR delayed the aggregation process slightly more than LP2. Taken together with the CZE data, these results point out two complementary inhibitory contributions: LP2 interacts efficiently with monomeric hIAPP, likely through noncovalent complexation, whereas nJNR contributes to partially stabilizing the monomeric form. Nonetheless, neither system alone sufficiently prevents the conversion of hIAPP monomers into fibril‐forming oligomers. This is consistent with the persistence of intermediate species with LP2 and their partial suppression with nJNR as observed in the CZE experiments. In sharp contrast, JNR‐LP2 (Figure 6A, orange curve) demonstrated a significantly stronger inhibitory effect, reducing the final ThT fluorescence intensity by 40%. This likely reflects a substantial suppression of β‐sheet‐rich fibril formation, corroborating CZE results showing a complete absence of oligomeric intermediates under JNR‐LP2 treatment. JNR‐LP2 also caused the most pronounced kinetic delay, both in lag phase and half‐time of aggregation (∼11%), reinforcing its superior efficacy in modulating the aggregation landscape of hIAPP.

**TABLE 1:** Effects of LP2, JNR‐LP2, and nJNR on hIAPP fibrillation as assessed by ThT fluorescence spectroscopy.

Sample	**Inhibitor:hIAPP** **molar ratio**	***t***_**1/2**_, **h**	**Fluorescence intensity** **at the plateau, RFU**
hIAPP	0	2.64 ± 0.04	3305 ± 7
hIAPP + nJNR	N.A.	2.85 ± 0.02	2961 ± 8
hIAPP + JNR‐LP2	1:1	2.92 ± 0.04	1949 ± 4
hIAPP + LP2	1:1	2.72 ± 0.05	2282 ± 8

*Note*: Values represent the mean ± standard deviation obtained from Boltzmann sigmoidal fits of ThT fluorescence curves from three independent experiments (Figure 6A).

Abbreviation: N.A.: nonapplicable.

It should be highlighted that although nJNR have a slight inhibitory role on fibril formation, the inhibition of fibril formation by JNR‐LP2 is much stronger than that of nJNR and also stronger than that of LP2 alone, revealing a synergistic effect when LP2 ligands are multiply attached to JNR particles. The conjugation appears to enhance both the binding efficiency to monomeric hIAPP and the sequestration of aggregation‐prone species, resulting in more sustained inhibition than either component alone. This synergy likely arises from spatially organized interactions at the NP interface, which not only delay but fundamentally reroute the aggregation process, as revealed by the combined ThT and CZE analyses. The present experimental design does not allow explicit deconvolution of primary and secondary nucleation contributions. Nevertheless, the complete suppression of soluble oligomeric intermediates observed by CZE under JNR‐LP2 treatment strongly suggests that the sequestration of monomeric hIAPP effectively depletes the pool of aggregation‐competent species required for both nucleation pathways. Indeed, secondary nucleation is itself monomer‐dependent [50]; the near‐total stabilization of monomeric hIAPP by JNR‐LP2 is expected to indirectly quench fibril surface‐catalyzed nucleation as well. Cell toxicity studies related to their exposure to hIAPP oligomers, conducted with or without LP2‐JNR, could serve as a way to support this hypothesis and represent the last step of our study (Section 2.4).

### 2.3 Affinity and Selectivity of JNR‐LP2 Toward hIAPP

Building on our previous findings demonstrating that JNR‐LP2 strongly inhibits hIAPP aggregation by stabilizing monomeric forms and suppressing transient oligomeric intermediates, we sought to further elucidate the molecular underpinnings of this inhibitory effect. In particular, we aimed to characterize the interaction between JNR‐LP2 and hIAPP at the molecular level and assess the selectivity of this interaction concerning other amyloidogenic proteins. To this end, we conducted two complementary experiments. First, we employed FACCE to quantify the binding parameters between hIAPP and both free LP2 and JNR‐LP2. This technique enabled us to evaluate the extent to which surface conjugation of LP2 to the JNR scaffold enhances its interaction with hIAPP, likely through multivalent and interfacial effects. Second, to investigate the target specificity of JNR‐LP2, we examined its impact on the fibrillation behavior of an unrelated amyloidogenic protein, Tau, under comparable in vitro conditions. These experiments were designed to distinguish whether the inhibitory activity of JNR‐LP2 is broadly antiamyloid or instead arises from a selective recognition mechanism specific to hIAPP.

#### 2.3.1 Interaction of LP2 and JNR‐LP2 Toward hIAPP

**FIGURE 7:**
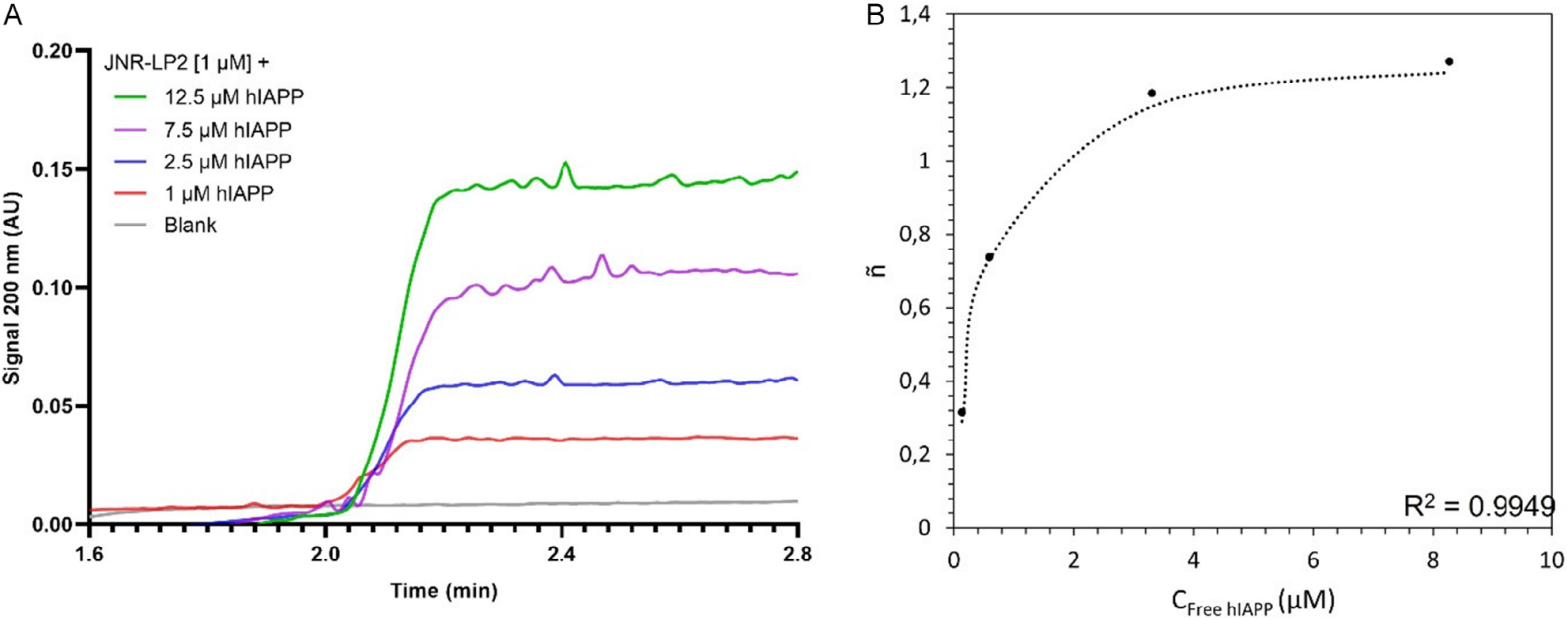
FACCE analysis of hIAPP–JNR‐LP2 interactions. (A) Representative FACCE electropherograms of free hIAPP after incubation with 1 µM JNR‐LP2 (the molar concentration indicated here corresponds to that of LP2 ligand, not of JNRs) and varying hIAPP concentrations. Each trace is the average of three independent experiments, with a standard deviation < 0.006 for plateau height values. (B) Adsorption isotherms derived from FACCE measurements, fitted to the Langmuir binding model. JNR‐LP2 concentration was fixed at 1 µM, while hIAPP ranged from 1 to 12.5 µM. Data are presented as mean ± SD (*n* = 3); error bars are present but not visually discernible due to their small magnitude.

To determine whether the inhibitory effect of JNR‐LP2 on hIAPP aggregation results from direct molecular interactions, we developed a FACCE method to measure both the binding constant (*k*) and binding stoichiometry (*n*) of hIAPP with either free LP2 or JNR‐LP2. FACCE is a quantitative affinity capillary electrophoresis method that estimates binding parameters based on differences in the electrophoretic migration between free and bound analytes. The principle involves preincubating the analyte of interest (hIAPP) with potential binding partners, followed by the selective and continuous electrokinetic injection of the free, unbound form of hIAPP for quantification. The schematic setup representation is shown in Figure S13A. This configuration was selected considering the very low electrophoretic mobility of LP2‐JNR (see Section 2.1.2.2). To ensure accuracy and reproducibility, the inner surface of the silica capillary was coated with 0.2% polybrene to prevent hIAPP adsorption and to enable selective injection of free hIAPP into the system. Method validation demonstrated high precision (RSD < 1%) and good linearity (R^2^ = 0.996) (Figure S13B,C). For binding experiments, hIAPP was incubated at increasing concentrations with either LP2 or JNR‐LP2 (1 µM fixed ligand concentration) at 37°C for 1 h under gentle agitation to allow equilibrium binding. The resulting electropherograms consistently showed a single plateau corresponding to free hIAPP. Interestingly, while a shift in the migration time of hIAPP was observed in both cases, 1.7 min with LP2 and 2.5 min with JNR‐LP2, the electropherogram profiles diverged significantly. In the presence of LP2, the plateau height of hIAPP increased slightly relative to the control, indicating that no appreciable binding occurred (Figure S14). Conversely, incubation with JNR‐LP2 led to a pronounced decrease in the plateau intensity of free hIAPP (Figure 7A), indicative of complex formation and reduced availability of unbound peptide. This allowed the determination of binding parameters using the Langmuir nonlinear model, yielding a binding constant of *k* =  (2.14 ± 0.2) × 10^6^ M^−1^ and a binding stoichiometry of *n* = 1.31 ± 0.08, with a strong correlation coefficient (Figure 7B). Here, we stress that the stoichiometry indicated corresponds to the hIAPP molecules:LP2 ligands stoichiometry. As each JNR‐LP2 nanocylinder bears ∼15 LP2 ligands, the hIAPP molecule to JNR‐LP2 nanocylinders is much higher (∼20:1), exemplifying the ability of JNR‐LP2 to capture several hIAPP molecules. Importantly, when FACCE was performed with nJNR at the same mass concentration as JNR‐LP2, no measurable interaction with hIAPP was detected, indicating negligible affinity. This confirms that the binding observed with JNR‐LP2 is specifically attributable to the presence of LP2 ligands on the nanorod scaffold. Together, these findings demonstrate a high‐affinity interaction between hIAPP and JNR‐LP2, in line with its capacity to bind and inhibit hIAPP oligomerization.

#### 2.3.2 Specificity of JNR‐LP2 for hIAPP

Building on the strong inhibitory effect of JNR‐LP2 on hIAPP aggregation, we next sought to determine whether this nanostructure exerts similar activity against other amyloidogenic proteins, in order to evaluate the specificity of its interaction and inhibition. To this end, we investigated its effect on the in vitro aggregation of Tau, a structurally unrelated amyloidogenic protein involved in Alzheimer's disease pathology [51]. As no CZE‐based method has been reported until now for monitoring Tau aggregation in vitro, we employed the ThT fluorescence assay to assess fibril formation in the presence of LP2, nJNR, and JNR‐LP2.

At a 1:1 molar ratio, free LP2 not only failed to inhibit Tau aggregation but also modestly favored the process, leading to both an earlier onset of fluorescence and an increase in the final fluorescence intensity (Figure 6B). This behavior stands in stark contrast to its inhibitory effect on hIAPP aggregation and underscores the high degree of target selectivity in LP2's mechanism of action. Consistent with this selectivity, LP2 has previously been shown to lack inhibitory activity against amyloid‐β42 aggregation [52]. Notably, this selective behavior was preserved upon conjugation to the nanostructure: JNR‐LP2 exhibited no inhibitory effect on Tau fibrillation under identical conditions. Furthermore, a slight acceleration of Tau aggregation was observed in the presence of nJNR, possibly due to nonspecific interactions with heparin [53], the anionic cofactor routinely employed to trigger Tau fibrillation in vitro.

Collectively, these results demonstrate that the inhibitory effect of JNR‐LP2 is selective for hIAPP and is not broadly applicable to other amyloidogenic proteins such as Tau. This highlights the critical role of molecular recognition in developing selective and effective antiamyloid nanotherapeutics.

### 2.4 JNR Restores Cell Viability by Mitigating hIAPP‐Induced Cytotoxicity

hIAPP oligomers are widely recognized as the principal cytotoxic species contributing to pancreatic β‐cell dysfunction, primarily through mechanisms involving membrane disruption, oxidative stress, and mitochondrial impairment [10]. To evaluate both the biocompatibility and therapeutic potential of our designed JNRs, we conducted two complementary MTT (3‐(4,5‐Dimethylthiazol‐2‐yl)‐2,5‐Diphenyltetrazolium Bromide)–based cell viability studies using the INS‐1 pancreatic β‐cell line.

First, we assessed the intrinsic cytotoxicity of JNR‐LP2, its molecular precursor LP2, the nonfunctional nJNR particles, and resveratrol, a polyphenolic antioxidant commonly used as a benchmark for cytoprotection [54]. LP2 and resveratrol were tested at 1 µM for 24 h, while nJNR was evaluated at the same mass concentration as JNR‐LP2 to ensure comparability. Under these conditions, all compounds exhibited excellent biocompatibility, maintaining cell viability above 95%, comparable to untreated controls (Figure S15). These results validate their safety profiles and support their suitability for subsequent therapeutic testing.

Next, we examined the protective efficacy of these compounds against hIAPP‐induced cytotoxicity, a pathological hallmark driven by the accumulation of early oligomeric intermediates. As expected, treatment of INS‐1 cells with hIAPP at increasing concentrations (1, 8, and 12.5 µM) resulted in a pronounced, dose‐dependent reduction in cell viability, with values falling to 57% at the highest concentration (Figure S16). This decline reflects the well‐characterized cytotoxicity of hIAPP oligomers, mediated by the disruption of membrane integrity and induction of cellular stress responses they provoke [43].

**FIGURE 8:**
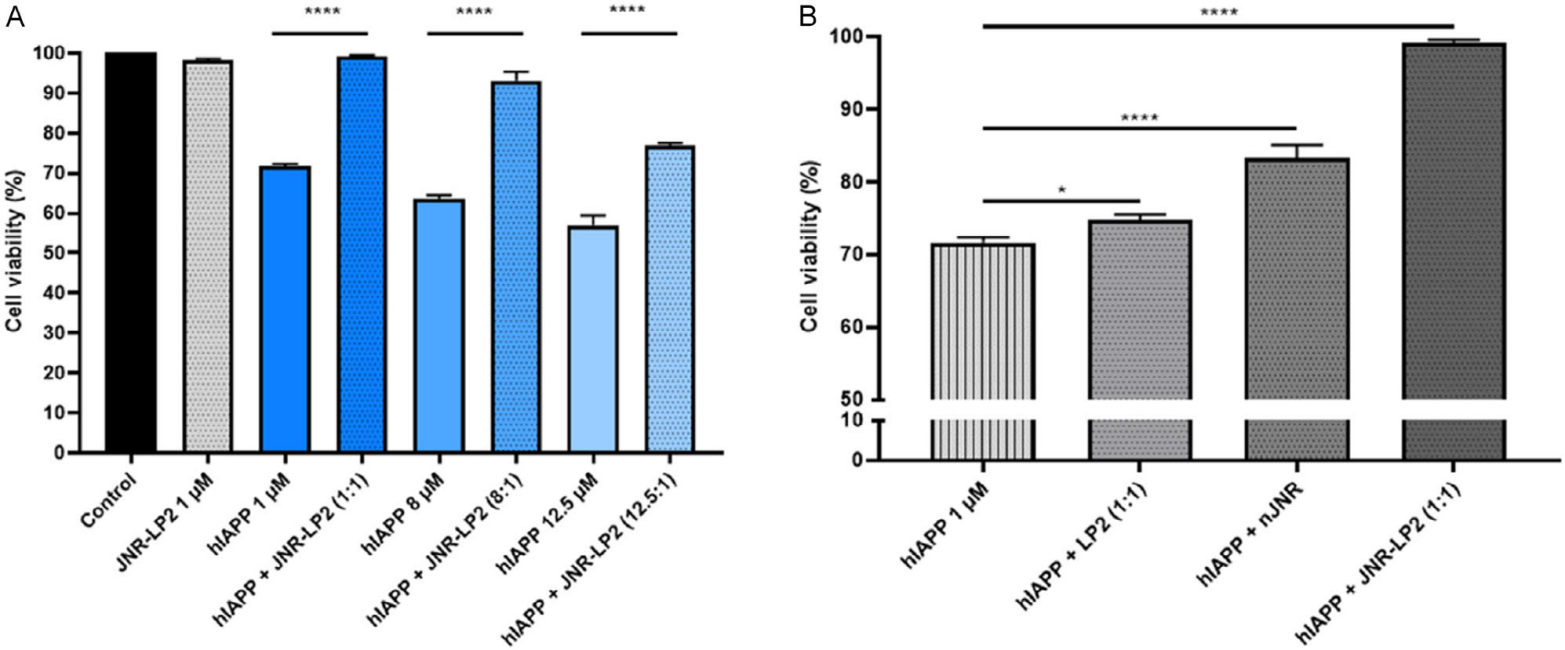
Protective effects of JNR‐LP2 and related compounds against hIAPP‐induced cytotoxicity in INS‐1 pancreatic β‐cells. Cell viability was assessed by MTT assay after exposure to hIAPP at the indicated concentrations, alone or coincubated with inhibitors. (A) Dose‐dependent restoration of viability by JNR‐LP2 at hIAPP:LP2 ligands molar ratios of 1:1, 8:1, or 12.5:1. (B) Comparative effects of LP2, nJNR, and JNR‐LP2 on cells treated with 1 µM hIAPP. Data represent mean ± SD (*n* = 3). Statistical significance was determined by one‐way ANOVA with Dunnett's post hoc test, comparing each cotreatment to the corresponding hIAPP‐only control (*p < 0.05; ****p < 0.0001).

Strikingly, coincubation with JNR‐LP2 at a 1:1 molar ratio (hIAPP: LP2 ligands) fully abrogated hIAPP‐induced toxicity, restoring cell viability to 99% (*p* < 0.0001 vs. hIAPP alone). Even under more challenging conditions where hIAPP was in molar excess (8:1 and 12.5:1), JNR‐LP2 maintained a strong protective effect, preserving 93% and 77% viability, respectively (Figure 8A). This dose‐resilient cytoprotection highlights the great therapeutic potential of the JNR‐LP2.

To contextualize the efficacy of JNR‐LP2, we compared its performance under identical conditions with resveratrol, known as a reference, and LP2. While resveratrol improved, as expected, cell viability to 79% at a 1:1 molar ratio with hIAPP, JNR‐LP2 consistently outperformed it across all tested conditions (Figure S17). Similarly, free LP2 alone restored viability to 75% (Figure 8B), comparable to resveratrol. These results suggest that the molecular pharmacophores themselves are moderately protective but do not reach the potency of the nanostructured formulation JNR‐LP2. Eventually, when comparing the protective efficacy of JNR‐LP2 with that of nJNR under identical conditions (Figure 8), JNR‐LP2 remained significantly (*p* < 0.0001) more effective than nJNR, highlighting the critical role of the LP2 ligand in enhancing the nanoinhibitor's functionality.

An important consideration for any nanotherapeutic targeting membrane‐active amyloid species is the potential for unintended interactions with cellular membranes. The excellent biocompatibility observed for JNR‐LP2 (>95% cell viability at therapeutic concentrations) suggests minimal membrane perturbation, likely attributable to the hydrophilic PDMAc polymer backbone and the modest negative surface charge (*ζ* = −13.6 mV) that prevents strong electrostatic interactions with negatively charged phospholipid headgroups typical of mammalian cell membranes. Moreover, the anisotropic architecture of JNRs, with LP2 ligands concentrated on a single face, may reduce nonspecific membrane binding compared to isotropically functionalized NPs by limiting the contact area available for membrane interaction. Importantly, the protective mechanism of JNR‐LP2 operates through solution‐phase sequestration of hIAPP monomers and prevention of oligomerization rather than direct membrane competition, thereby preserving normal membrane physiology while neutralizing amyloid toxicity. Future studies employing model membrane systems (e.g., supported lipid bilayers and liposomes) [55] will be valuable to quantitatively assess JNR–membrane interactions and further validate the membrane‐safe profile of this approach.

Overall, these results position JNR‐LP2 as the most effective formulation, outperforming both free LP2 and nJNR. The superior and dose‐resilient cytoprotection of JNR‐LP2 likely arises from its tailored ability to selectively capture hIAPP monomers, avoiding oligomers formation, thereby preventing their interaction with cellular membranes and halting the cascade of amyloid‐induced cytotoxic events. The combination of excellent biocompatibility, strong inhibitory potency, and nanostructure‐enabled activity highlights JNR‐LP2 as a promising candidate for further development in combating amyloid‐driven β‐cell dysfunction.

### 2.5 Discussion

Several classes of nanomaterials capable of inhibiting hIAPP aggregation have been reported to date, although their mechanisms of action remain incompletely understood and are highly dependent on their physicochemical properties such as composition, surface chemistry, and morphology. For instance, PAMAM‐OH dendrimers have been shown to inhibit hIAPP aggregation via nonspecific electrostatic and hydrophobic interactions without involving sequence‐selective recognition [26]. Discrete molecular dynamics (DMD) simulations suggested that these dendrimers bind preferentially to the amyloidogenic region of hIAPP (residues 22–29), thereby disrupting interpeptide hydrogen bonding and hydrophobic interactions that are critical for self‐assembly. Similarly, carbon‐based nanomaterials such as fullerenes and their derivatives have demonstrated inhibitory activity against hIAPP aggregation [24]. Replica exchange molecular dynamics simulations revealed that pristine C60 primarily interacts through hydrophobic and π–π stacking interactions, whereas hydroxylated fullerenes additionally engage in hydrogen bonding, leading to the stabilization of disordered conformations and prevention of β‐sheet formation.

In the present study, we focused on the early stages of hIAPP aggregation. Using complementary techniques such as CZE and ThT fluorescence assays, we demonstrate that JNR‐LP2 stabilizes monomeric hIAPP and suppresses transient oligomer formation, thereby inhibiting nucleation and delaying fibril growth (Figures 3 and 6). This behavior is consistent with a solution‐phase sequestration mechanism driven by multivalent interactions. The high binding affinity (*k* = 2.14 × 10^6^ M^−1^, Figure 7), combined with the multivalent presentation of LP2 ligands, likely enhances this effect through cooperative binding and local concentration.

Beyond early‐stage inhibition, recent studies have shown that certain nanomaterials can also destabilize preformed amyloid assemblies. For example, charged GQDs disrupt hIAPP fibrils by reducing β‐sheet content, altering the hydrophobic core, and interfering with interstrand hydrogen bonding [56]. Although not directly addressed here, the multivalent and directional presentation of β‐hairpin mimetic LP2 motifs on the Janus surface may enable interaction with exposed β‐sheet edges or fibril interfaces, potentially interfering with elongation or locally destabilizing protofibrillar structures. Future studies combining high‐resolution imaging and molecular simulations will help clarify this potential.

An important consideration for nanomaterials targeting membrane‐active amyloid species is the risk of unwanted interactions with cellular membranes [57]. For instance, hydrophobic nanomaterials, such as graphene‐based systems, can induce membrane stress through lipid extraction and membrane disruption [58]. In contrast, for peptide‐modified gold nanoclusters, the presence of the negatively charged lipid membrane partly cancels the electrostatic interactions between gold nanoclusters and hIAPP and increases the hydrophobic interaction between the apolar residues in the peptide ligand of the nanocluster and their homologous residues in the core region of hIAPP, thereby protecting cell membranes from damage by hIAPP [59]. JNR‐LP2 is expected to mitigate negative effects on membranes due to its hydrophilic PDMAc backbone, modest negative surface charge (*ζ* = –13.6 mV), and anisotropic Janus architecture, which limits membrane contact. Consistently, high cell viability (>95%, Figure S15) indicates minimal membrane perturbation under the tested conditions. This is further supported by the anticipated mechanism of JNR‐LP2, involving solution‐phase sequestration of hIAPP rather than direct membrane interaction. Future studies employing model membrane systems (e.g., supported lipid bilayers, liposomes, or giant unilamellar vesicles) will be valuable to detect potential JNR–membrane interactions and further validate the membrane‐compatible profile of this approach [55].

Compared with previously reported nanomaterials, JNR‐LP2 offers several distinctive advantages. First, it exhibits a high degree of sequence selectivity, as demonstrated by the absence of inhibitory activity toward Tau aggregation. This distinguishes it from many nanomaterials that rely primarily on nonspecific interactions. Among targeted approaches, nanobody–gold NP conjugates represent a notable example capable of selectively binding hIAPP monomers or oligomers and redirecting them into nonamyloid complexes [30]. In that system, multivalency and confinement effects were proposed to enhance inhibitory activity. Similarly, in our system, the presence of multiple LP2 ligands on a single nanorod likely contributes to a strong synergistic effect through multivalent binding and local concentration enhancement. Second, JNR‐LP2 effectively targets early oligomeric species, which are widely recognized as the most cytotoxic intermediates in amyloid diseases. This contrasts with many nanomaterials that primarily affect late‐stage fibrillation. In previous work [35], LP2 was shown to form complexes with both monomeric and oligomeric hIAPP species, supporting its role in early‐stage inhibition. Third, the Janus architecture provides a unique level of modularity, enabling asymmetric and independent functionalization of both faces, which opens perspectives for dual‐targeting strategies that remain largely inaccessible with isotropic NPs. To the best of our knowledge, very few Janus nanomaterials have been explored in the context of amyloid inhibition. A notable example is the use of β‐casein‐coated iron oxide Janus NPs, which have been reported to inhibit the aggregation of amyloid β, hIAPP, and α‐synuclein [19]. In that system, DMD simulations suggested that β‐casein adsorbed at the NP surface can encapsulate amyloidogenic peptides, thereby interfering with their aggregation pathways. However, despite these promising results, issues related to sequence specificity, mechanistic control, and biocompatibility remain insufficiently addressed.

In this context, the present work introduces a fundamentally different approach based on a fully polymeric and anisotropic platform, combining sequence‐specific recognition through LP2 ligands with controlled multivalent presentation. This strategy highlights the potential of rationally designed Janus nanostructures to achieve selective and mechanism‐driven modulation of amyloid aggregation.

## 3 Experimental Section

### 3.1 Chemicals and Reagents

hIAPP (TFA salt) was purchased from Bachem (Bubendorf, Switzerland). Sodium hydroxide (NaOH), sodium chloride (NaCl), ammonium acetate, DMSO, hexafluoroisopropanol (HFIP), methanol, hexadimethrine bromide (polybrene, MW 4000–6000 g/mol), and ThT were all purchased from Sigma–Aldrich (Saint‐Quentin Fallavier, France). One‐micrometer PTFE syringe filters were purchased from Merck (Darmstadt, Germany). *N*, *N*‐dimethylacrylamide (DMAc, 99%, Aldrich) was passed through basic aluminum oxide to remove the inhibitor. 2,2′‐Azobis(2‐methylpropionitrile) (AIBN, 98%, Sigma–Aldrich) was recrystallized from methanol. DMSO (≥ 99.9%, Fisher Chemical), *N*, *N*‐dimethylformamide (DMF, 99.8%, extra dry over molecular sieve, Thermo Scientific), 1,3,5‐trioxane (99.5%, Acros Organics), EDC (98%, ABCR), ethyl cyanohydroxyiminoacetate (Oxyma Pure, Novabiochem), 4‐dimethylaminopyridine (DMAP, ≥99%, Sigma–Aldrich), and ACPA (98%, Thermo Scientific) were used as received. Toluene was obtained from a solvent purification system (MBraun SPS). The trisurea‐functional RAFT agents, 48‐CTA and 84‐CTA, were synthesized as reported previously [31]. All solutions were prepared with ultrapure water from a MilliQ system (Merck Millipore, Fontenay‐sous‐Bois, France) and filtered through a 0.22 µm nylon filter (VWR, Fontenay‐sous‐Bois, France).

### 3.2 Synthesis of the hIAPP Ligand

The LP2 ligand was synthesized and purified according to the procedure described in the literature [35].

### 3.3 Preparation of the JNR

#### 3.3.1 Polymerization of DMAc

##### 3.3.1.1 Synthesis of 48‐PDMAc_80_

Following the previously published procedure [31], a 250 mL round‐bottom flask equipped with a magnetic stirring bar was charged with DMAc (4.0 g, 40.4 mmol, 114 eq), 48‐CTA (280 mg, 0.354 mmol, 1 eq), DMSO (197 mL), and trioxane (145 mg, 1.61 mmol, 4.55 eq). Then, the mixture was heated at 65°C for 30 min to obtain a homogeneous solution and was bubbled with N_2_ before the injection of 1 mL of a 5.81 mg/mL stock solution of AIBN in DMSO (5.81 mg AIBN, 0.1 eq). The solution was bubbled with N_2_ for another 10 min. After 160 min, the flask was plunged into an ice‐water bath, and the cap was opened after several minutes. Sixty‐eight percent for the final conversion was determined by ^1^H‐NMR (relative integration of the internal reference peak at 5.0 ppm and CH_2_ = CH peak at 5.5 ppm). The polymer solution was purified by dialysis with a 1 kDa membrane and a further freeze‐drying step to give a yellow powder (2.6 g, 87%). A number‐average degree of polymerization (DP_*n*_) of 80 was determined by ^1^H‐NMR of the final polymer. The number‐average molar mass (*M*_*n*_) and molar mass dispersity (*Ð*) were determined by SEC (*M*_*n*_ (SEC) = 9.7 kg/mol, *Ð* = 1.09).

##### 3.3.1.2 Synthesis of 84‐PDMAc_60_

Following the previously published procedure [31], a 500 mL round‐bottom flask with a stir bar was charged with 8/4‐CTA (353 mg, 0.446 mmol, 1 eq), DMSO (268 mL), and DMAc (6.2 g, 62.5 mmol, 140 eq) and then degassed by bubbling argon gas at 25°C for 45 min. The mixture was then heated to 70°C to obtain a homogeneous solution and equipped with a balloon filled with argon. Then, 10 mL of dry toluene (used as an internal standard) and 1 mL of AIBN (0.045 mmol, 0.1 eq) from a degassed stock solution of 7.3 mg/mL in DMSO were added to the solution under argon. Aliquots of 0.7 mL were withdrawn periodically from the reaction mixture to determine the conversion by ^1^H‐NMR and molar mass by SEC. After a 48% conversion (approximately 1.5 h), the reaction was quenched by being exposed to air and cooled with liquid nitrogen. The reaction mixture was transferred at room temperature to a 3.5 kDa RC dialysis tubing and dialyzed against deionized water (DIH_2_O) for a minimum of three times before being freeze‐dried to obtain a yellow powder (2.4 g, 81% yield). *DP*_*n*_ = 60 was determined by ^1^H‐NMR of the final polymer (*M*_*n*_ (SEC) = 7.5 kg/mol, *Ð* = 1.09).

#### 3.3.2 Functionalization with Grafting of the hIAPP Ligand

##### 3.3.2.1 Synthesis of 48‐PDMAc_80_‐COOH

A 50 mL round‐bottom flask equipped with a stir bar was charged with 2.03 g of ACPA (7.2 mmol, 60 equiv.), dissolved with 10 mL DMSO, sealed with a septum, and degassed with argon for 30 min. In a vial equipped with a pierceable cap, 1 g of 48‐PDMAc_80_ (8.3 kg mol^−1^, 120.5 µmol, 1 equiv.) was dissolved in 15 mL of DMSO and then degassed with argon for 15 min. The solution of ACPA was heated to 65°C, whereupon reaching the temperature, the 48‐PDMAc_80_ solution was added dropwise via cannula transfer at 1 mL/min under argon. The reaction was left to react for 8 h before being charged with an additional 1.35 g of ACPA (4.81 mmol, 40 equiv.) under argon purge and then left to react overnight (16 h). The following day, the reaction was charged again with 1.35 of ACPA (4.81 mmol, 40 equiv.) under argon purge and then left to react for another 8 h. The reaction was quenched by opening the round‐bottom flask and cooling it in an ice bath. The reaction was warmed to 25°C, transferred to a 1 kDa RC dialysis tubing, and dialyzed against MeOH (three times) and H_2_O (three times) and finally freeze‐dried (760 mg, 76% yield) (*M*_*n*_ (SEC) = 12.3 kg/mol, *Ð* = 1.15).

##### 3.3.2.2 Synthesis of 48‐PDMAc_80_‐LP2

In a dry 25 mL round‐bottom flask equipped with a stir bar, 125 mg (8.3 kg/mol, 15 µmol, 1 eq.) of 48‐PDMAc_80_‐COOH was dissolved in 5.5 mL of dry DMF. To the solution, ethyl cyanohydroxyiminoacetate (Oxyma, 21.3 mg, 150 µmol, 10 eq.), DMAP (18.3 mg, 150 µmol, 10 eq.), and EDC (29 mg, 150 µmol. 10 eq.) were added and left to react at room temperature. After 1 h, LP2 (15 mg, 13.5 µmol, 0.9 eq.) was added to the solution and left to react for 24 h at room temperature. The reaction was transferred to a 3.5 kDa RC dialysis tubing, dialyzed against MeOH (5 times) and H_2_O (3 times). The final white polymer (33 mg, 26% yield) was obtained after lyophilization (*M*_*n*_ (SEC) = 13.2 kg/mol, *Ð* = 1.11).

#### 3.3.3 JNRs CoAssembly

##### 3.3.3.1 CoAssembly of nJNRs

Nonfunctionalized JNR (nJNR) was formed by coassembly of 48‐PDMAc_80_ + 84‐PDMAc_60_ in aqueous solution following an already published so‐called “DMSO‐route” [32, 60]. Briefly, 48‐PDMAc_80_ and 84‐PDMAc_60_ were dissolved in DMSO at a total polymer concentration *C*_0_ = 90 g/L. Then, water was added at a constant flow rate, *R* = 0.5 mL/h, and at a temperature *T* = 20°C until a final concentration of *C*_f_ = 0.9 g/L and a DMSO/water content of 1/99 by volume was reached.

##### 3.3.3.2 CoAssembly of LP2‐Functionalized JNR (JNR‐LP2)

JNR‐LP2 was obtained by coassembly of a mixture of LP2‐functionalized and nonfunctionalized PDMAc arms in aqueous solution, following the same “DMSO‐route” protocol used for nJNRs. Specifically, one equivalent of a 9:1 molar ratio of 48‐PDMAc_80_ and 48‐PDMAc_80_‐LP2 (note that only 30 mol% of 48‐PDMAc_80_‐LP2 actually bear a LP2 ligand, see part 2.1.1) was mixed with one equivalent of 84‐PDMAc_60_ in DMSO at a total polymer concentration *C*_0_ = 90 g/L. Coassembly was triggered by the gradual addition of water at a constant flow rate of 0.5 mL/h and at a temperature of 20°C, until a final polymer concentration *C*_f_ = 0.9 g/L and a DMSO/water content of 1/99 by volume were reached.

For both nJNR and JNR‐LP2, a final purification step was carried out by dialysis against water using a 1 kDa molecular weight cutoff membrane, in order to remove residual DMSO and obtain DMSO‐free dispersions. The dialysis medium was replaced (every 2 h initially) over a 24‐h period. Following dialysis, UV‐vis spectroscopy (absorbance at 309 nm) was used to determine the exact polymer concentration.

### 3.4 Polymer and JNR Physicochemical Characterization

#### 3.4.1 Polymer Characterization

##### 3.4.1.1 NMR

^1^H‐NMR spectra were recorded at 27°C with a Bruker 400 or 600 MHz spectrometer. Deuterated DMSO (DMSO‐d6, 99.9%, Innova‐chem) and methanol (CD_3_OD, 99.8%, Innova‐Chem) were used as NMR solvents.

##### 3.4.1.2 SEC

Polymers were analyzed by SEC in DMF (+ LiBr, 1 g/L). The analyses were performed at 60°C, at a flow rate of 0.8 mL/min, and a polymer concentration of 5 g/L after filtration through a 0.22 µm membrane. The SEC analyses were carried out on two PSS GRAM 1000 Å columns (8 × 300 mm; separation limits: 1–1000 kg/mol) and one PSS GRAM 30 Å (8 × 300 mm; separation limits: 0.1–10 kg/mol) coupled with a differential RI detector and a UV detector. The OmniSEC 5.12 software was used for data acquisition and data analysis. Molar masses (*M*_*n*_, *M*_w_) and dispersity (*Ð* = *M*_w_/ *M*_*n*_) were calculated from the conventional RI calibration based on PMMA (Poly (methyl) methacrylate) standards (from Polymer Standard Services).

##### 3.4.1.3 UV Spectroscopy

UV‐vis spectra of the polymer solutions in methanol with concentration of 1 g/L were recorded on a JASCO V‐670 spectrometer.

#### 3.4.2 JNR Physicochemical Characterization

##### 3.4.2.1 Cryo‐TEM

JNR samples were analyzed by cryo‐TEM without prior filtration. For sample preparation, a droplet of the aqueous solution was deposited onto a Quantifoil‐coated carbon grid. Excess liquid was removed either manually or using a CryoPlunge 3 system (Gatan), and the resulting thin liquid film was rapidly vitrified by plunging into liquid ethane. It is important to note that this standard blotting procedure can induce local concentration variations on the grid due to capillary flow and liquid removal, and therefore the particle densities observed in the cryo‐TEM images should not be interpreted as representative of the actual concentration in solution. Imaging was performed at −180°C using a JEOL JEM‐2100 LaB_6_ electron microscope operated at 200 kV under low‐dose conditions (∼10 electrons Å^−2^ s^−1^). Digital micrographs were acquired with a Gatan Ultrascan 1000 2k × 2k CCD camera. Multiple regions of each grid were examined at various magnifications to ensure the morphological consistency and representativity of the nanorods presented in this study. For size analysis, the diameters of all nanorods within defined sections of representative micrographs were measured, totaling 60–100 nanorods per sample.

##### 3.4.2.2 SLS

SLS measurements were done at 20°C on an ALV‐CGS3 system operating with a vertically polarized laser with wavelength *λ *= 633 nm to cover a 2.8 × 10^6^ to 2.6 × 10^7^ m^−1^ *scattering vector* (*q*)‐range, which is defined as Equation (1):

(1) $$q=\frac{4\pi n\sin\;\frac{\theta }{2}}{\lambda }$$

where *θ* is the angle of observation, and *n* = 1.33 is the RI of the solvent.

The solutions were filtered using 1 μm pore size Whatman filters before SLS analysis. The normalized scattered intensity measured by SLS is represented by the Rayleigh ratio determined following Equation (2):

(2) $$R_{\theta }=\frac{I_{\text{solution}}\left(\theta \right)-I_{\text{solvent}}\left(\theta \right)}{I_{\text{toluene}}\left(\theta \right)}.{\left(\frac{n_{\text{solvent}}}{n_{\text{toluene}}}\right)}^{2}.\;R_{\text{toluene}}$$

where *I*_solution_, *I*_solvent_, and *I*_toluene_ are the average intensities scattered, respectively, by the solution, the solvent, and the reference (toluene) at a given angle *θ*, *n*_solvent_ = 1.33 and *n*_toluene_ = 1.50 are the respective RIs of the solvent and of toluene, and *R*_toluene_ = 1.35·10^−5^ cm^−1^ is the Rayleigh ratio of toluene for a wavelength *λ* = 633 nm. For water solutions containing small amounts of DMSO, *n*_solvent_ was taken equal to that of water [31].

##### 3.4.2.3 DLS

The normalized electric field autocorrelation function *g*_1_(*q*, *t*) was measured by DLS, from which the relaxation time *τ* can be extracted assuming a distribution *A*(*τ*) of different relaxation times *τ*. The REPES routine was used to obtain *A*(*τ*) without assuming a specific shape for the distribution (Equation (3)).

(3) $$\left(t\right)=\int A(\tau )\;e^{\left(-\frac{t}{\tau }\right)d\tau }$$

The apparent diffusion coefficient of the particles *D* can be determined from the average relaxation time *τ* of the relaxation mode as *D* = ⟨*τ* ^−1^⟩/*q* ^2^.

At the investigated concentration (∼1 g/L) and at sufficiently low q, the apparent diffusion coefficient of the particles D corresponds to the true diffusion coefficient of the particles and relates to their hydrodynamic radius, *R*_h_, according to Equation (4), as determined previously for similar JNR [32].

(4) $$R_{h}=\frac{kT}{6\pi \eta D}$$

where *k* = 1.38 × 10^−23^ J·K^−1^ is the Boltzmann constant, *T* is the absolute temperature, and *η* is the viscosity of the solvent.

##### 3.4.2.4 Zeta Potential Measurement

In order to remove spurious aggregates, JNR samples were subjected to a standardized pretreatment involving filtration through 1 µm PTFE membranes. This protocol ensured that measurements reflected the properties of dispersed, nonaggregated NPs. Then, the zeta potential of JNRs was measured using a Zetasizer Nano ZS (Malvern Panalytical, France). Measurements were performed in bicine buffer (100 mM, pH 7.4). Prior to measurement, samples were equilibrated at 25°C for 30 s to avoid convection‐induced artifacts. Bicine‐buffered conditions are suitable to assess the surface charge and colloidal stability of the JNRs, taking advantage of moderate conductivity to avoid electrokinetic artifacts typically encountered in low ionic strength media.

### 3.5 hIAPP and JNR Sample Preparation

#### 3.5.1 Preparation of hIAPP Solutions

hIAPP was first dissolved in pure HFIP at a concentration of 1 mM and incubated at room temperature for 1 h to disaggregate any preformed fibrils [61]. The HFIP was subsequently removed by evaporation under a gentle stream of dry nitrogen, followed by vacuum desiccation for a minimum of 3 h to ensure complete solvent removal. The resulting peptide film was aliquoted (39 µg per aliquot) and stored at –20°C until further use. For reconstitution, individual aliquots were dissolved in 50 mM ammonium acetate (pH 3.7) unless otherwise specified. Stock solutions were prepared at a final peptide concentration of 0.2 mM.

#### 3.5.2 Concentration of JNR‐LP2 and of the Reference Samples used for the Assays

To evaluate the effect of JNR‐LP2 on hIAPP oligomerization, fibrillation, and cell viability, experiments were performed with JNR‐LP2 as well as with nJNR and free LP2. For the sake of comparison, the total molar concentration of LP2 ligand in JNR‐LP2 solutions and in free LP2 solutions was kept constant. Typically, to achieve a 1 µM concentration of LP2, a mass concentration of JNR‐LP2 of 0.56 g/L was used. The number‐average molar masses of the 48‐PDMAc_80_ (*M*_n_ = 8.7 × 10^3^ g/mol, 45 mol% of the JNR‐LP2 solution), 48‐PDMAc_80_‐LP2 (*M*_n_ = 13.2 × 10^3^ g/mol, 5 mol% of the JNR‐LP2 solution, corresponding to 10 mol% relative to the 48‐PDMAc_80_ arms), and 84‐PDMAc_60_ (*M*_n_ = 6.7×10^3^ g/mol, 50 mol% of the JNR‐LP2 solution) is *M*_*n*,unimer_ = 7.9 × 10^3^ g/mol. Therefore, for [JNR‐LP2] = 0.56 g/L, the LP2 concentration is [JNR‐LP2].x_48‐PDMAc80‐LP2_.f/*M*_n,unimer_ = 1 µM, where x_48‐PDMAc80‐LP2_ = 5 mol% is the molar fraction of 48‐PDMAc_80_‐LP2 arms used to prepare JNR‐LP2 and *f* = 30% is the actual degree of functionalization of 48‐PDMAc_80_‐LP2 polymer arms with LP2 functions.

For nJNR solutions, which lack LP2 ligands, defining a comparable molar ratio in LP2 ligands was not feasible. Therefore, for control experiments involving nJNR, the same mass concentration of nJNR as that of the corresponding JNR‐LP2 samples was used.

### 3.6 In Vitro and Real‐Time Monitoring of hIAPP Aggregation

#### 3.6.1 CZE to Monitor hIAPP Oligomerization

CZE with UV detection (CZE‐UV) was performed using an MDQ instrument (SCIEX, Framingham, MA, USA) equipped with a UV detector set at 200 nm. Fused silica capillaries (50 µm i.d., 365 µm o.d.; Polymicro Technologies, Phoenix, AZ, USA) with a total length of 60 cm (effective length to the detector: 50 cm) were employed. The BGE consisted of 50 mM ammonium acetate adjusted to pH 3.7. Capillary coating and experimental conditions are the same as described in our previous work [39]. The coating efficiency was determined by measuring the residual EOF with DMSO as the neutral marker. hIAPP samples were prepared at concentrations of 100 or 12.5 µM. The ability of compounds to inhibit hIAPP oligomerization was assessed considering the persistence of the monomer peak by peak area calculations using the Karat32 software (Sciex).

#### 3.6.2 ThT Assay for Monitoring hIAPP and Tau Fibrillation

ThT fluorescence spectroscopy was used to monitor in vitro fibrillation of hIAPP and recombinant Tau protein (Wt‐Tau441). Measurements were performed using fluorescence plate readers equipped with 440 nm excitation and 480 nm emission filters (Fluostar Optima or Vantastar, BMG Labtech, Ortenberg, Germany). The ThT buffer for hIAPP experiments consisted of 10 mM Tris/HCl, 100 mM NaCl, and 10 µM ThT, adjusted to pH 7.4. For Tau assays, the buffer was 25 mM sodium phosphate (NaPi) at pH 6.8, containing 25 mM NaCl, 2.5 mM EDTA, and 25 µM ThT.

##### 3.6.2.1 hIAPP Fibrillation Assay

Aliquots of hIAPP (39 µg) were dissolved in 50 mM ammonium acetate buffer (pH 3.7) to a final concentration of 1 µM. Test compounds were then added directly into wells of black, flat‐bottom, 96‐well microtiter plates (Costar, Corning), followed by thorough mixing. The reaction volume in each well was adjusted to 100 µL using ThT assay buffer, consisting of 10 mM Tris/HCl, 100 mM NaCl, and 10 µM ThT, adjusted to pH 7.4. For inhibitor assays, compounds were tested at a final concentration of 1 µM: JNR‐LP2 and LP2 were applied at a 1:1 molar ratio relative to hIAPP, while nJNR control samples were supplemented with the same stock volume used for JNR‐LP2, thereby matching the particle mass concentration, as explained above. Negative control wells contained hIAPP in the ThT buffer without additional compounds. Fluorescence was monitored at 10‐min intervals over 42 h using a plate reader. Each condition was assayed in triplicate with the same batch of hIAPP to ensure reproducibility. Plates were sealed with optically transparent adhesive films to minimize evaporation. Aggregation kinetics were evaluated by fitting fluorescence intensity curves to a Boltzmann sigmoidal model using GraphPad Prism, from which the half‐aggregation time (*t*_1_/_2_) and the fluorescence plateau intensity (*F*) were determined as metrics of amyloid formation and compound efficacy.

##### 3.6.2.2 Tau Fibrillation Assay

Recombinant Tau protein was produced and purified according to a previously described protocol [62]. Stock Tau solutions (20 µM) were prepared in NaPi buffer. Test compounds JNR‐LP2 and LP2 were prepared as 2 mM stock solutions in MilliQ water. For each assay, Tau was diluted to 1 µM in NaPi buffer containing ThT (25 µM). Compounds were added to reach 1 µM for JNR‐LP2 and LP2 (1:1 molar ratio with Tau) or 5 µM for LP2 (5:1 ratio). nJNR control samples received the same stock volume as JNR‐LP2, matching the particle mass concentration, as explained above. Negative controls were prepared without test compounds. After a 60‐min incubation, fibrillization was initiated by adding heparin sodium salt (average molecular weight ∼18 kDa, Sigma–Aldrich H‐3149) to a final concentration of 0.1 µM. Measurements were performed in 384‐well black flat‐bottom plates (Optiplate 384, Revvity Health Sciences) with a 40 µL final volume per well. Fluorescence intensity was recorded for 24 h at 37°C, with continuous double‐orbital shaking at 300 rpm. Plates were sealed with optically transparent films. All assays were run in triplicate.

### 3.7 Analysis of JNR–hIAPP Interactions by FACCE

A fresh solution of hIAPP (25 µM) was prepared in ammonium acetate (50 mM, pH 3.7). Stock solutions of JNR‐LP2 and LP2 were diluted to the desired concentrations in the same buffer and incubated for 1 h at 37°C with hIAPP solution, resulting in final peptide concentrations ranging from 1 to 12.5 µM after incubation. All samples were prepared in triplicate. FACCE experiments were carried out on the same MDQ Instrument (SCIEX, Framingham, MA, United States) equipped with a UV detector as the one used for CZE and the same capillary preconditioning protocol. The sample was introduced from the short end (10 cm to the detector) of the polybrene‐coated capillary. The temperature of the capillary cartridge was set at 25°C. The capillary was rinsed between each run for 1 min with MilliQ water and 2 min with BGE. FACCE experiments were performed by applying a −20 kV voltage and a copressure of −30 mbar for 3 min to allow a continuous and electrokinetic injection of exclusively free hIAPP. All measurements were performed in triplicate.

The adsorption isotherms for the interaction between JNR‐LP2 or LP2 and hIAPP were established based solely on the free hIAPP concentrations determined experimentally by FACCE. The affinity constant (*k*) was determined by fitting the experimental adsorption isotherms to the Langmuir equation, assuming *n* independent binding sites of equal energy [63]. The parameters *n* and *k* were obtained by nonlinear curve fitting of the experimental data, with *n* corresponding to the maximum number of interacting sites at isotherm saturation.

### 3.8 Cell Culture and INS‐1 Viability

#### 3.8.1 Cell Culture

Rat insulinoma INS‐1 cells were used to assess cell viability. Cells were cultured in RPMI‐1640 medium supplemented with 10% fetal bovine serum, 2 mM L‐glutamine, 1 mM sodium pyruvate, 10 mM HEPES, 50 µM β‐mercaptoethanol, 100 U/mL penicillin, and 100 µg/mL streptomycin, and maintained at 37°C in a humidified atmosphere containing 5% CO_2_. Lyophilized hIAPP was first dissolved in HFIP to disaggregate preformed fibrils, and the solvent was evaporated under a gentle nitrogen stream. The resulting peptide film was freshly reconstituted in DMSO before each experiment to ensure complete dissolution; the final DMSO concentration in each well was kept below 1% (v/v). INS‐1 cells were seeded in 96‐well plates at 30,000 cells per well and allowed to adhere for 24 h before treatment with hIAPP (final concentrations: 1 µM, 8 µM, or 12.5 µM), either alone or in the presence of inhibitors (nJNR, JNR‐LP2, or LP2).

#### 3.8.2 Cell Viability Assay

Two experimental protocols were conducted to assess: (i) the intrinsic cytotoxicity of the tested compounds and (ii) their potential cytoprotective effects against hIAPP‐induced toxicity. In both cases, cell viability was evaluated using the MTT assay (3‐[4,5‐dimethylthiazol‐2‐yl]−2,5‐diphenyltetrazolium bromide), which measures the mitochondrial‐dependent reduction of MTT to insoluble formazan by metabolically active cells [64]. To assess whether the tested compounds affect cell viability in the absence of hIAPP, INS‐1 pancreatic β‐cells were treated for 24 h with either JNR‐LP2, free LP2, resveratrol (each at a final concentration of 1 µM), or nJNR at an equivalent volume to JNR‐LP2. In parallel, to investigate cytoprotective effects, cells were coincubated for 24 h with hIAPP at increasing concentrations, either alone or in combination with JNR‐LP2, LP2, or resveratrol. These compounds were added at molar ratios matching that of hIAPP (1:1, 8:1, or 12.5:1, hIAPP:compound). In the case of nJNR, the same weight concentration of JNR as that used for JNR‐LP2 was added. Following the 24‐h incubation period, 15 µL of MTT solution (5 mg/mL in PBS) was added to each well, and plates were incubated for an additional 2 h at 37°C. The medium was then carefully aspirated, and 200 µL of DMSO was added to solubilize the resulting formazan crystals. Absorbance was measured at 570 nm using a Multiskan MS microplate reader (Labsystems). Cell morphology was monitored throughout the assay using phase‐contrast microscopy to qualitatively assess treatment effects. Each condition was tested in eight replicate wells per experiment, and the entire experiment was independently repeated three times (*n* = 3). Data are expressed as mean ± standard deviation. Statistical analysis was performed using one‐way analysis of variance, followed by Dunnett's multiple comparison test (GraphPad Prism). Differences were considered statistically significant at *p* < 0.05.

## 4 Conclusion

This study introduces and validates JNRs functionalized with a β‐hairpin mimetic ligand (LP2) as a highly effective, selective, and modular nanoplatform for inhibiting the aggregation of hIAPP, a key pathogenic event in T2DM. Using an integrative strategy that combines advanced electrophoretic techniques (CZE, FACCE), fluorescence‐based aggregation assays (ThT), and cellular viability measurements, we show that JNR‐LP2 effectively interferes with hIAPP self‐assembly. Specifically, JNR‐LP2 not only delays fibril formation but also prevents the emergence of early soluble oligomers, which are widely recognized as the most cytotoxic amyloid species in the progression of T2DM [65]. Importantly, by suppressing hIAPP‐induced toxicity, JNR‐LP2 restores cellular viability, highlighting its potential as a protective agent against amyloid‐driven β‐cell dysfunction. Notably, this inhibition is achieved at substoichiometric concentrations, underscoring the enhanced efficacy imparted by multivalent ligand display and the unique anisotropic Janus architecture.

Mechanistically, JNR‐LP2 exerts a dual mode of action: it stabilizes monomeric hIAPP and disrupts the nucleation of oligomeric intermediates, thereby rerouting the aggregation pathway away from cytotoxic endpoints. The superior performance of JNR‐LP2 compared to its free ligand or nonfunctionalized counterparts highlights the critical role of nanoscale spatial organization in modulating protein–ligand interactions. Moreover, the specificity of JNR‐LP2 toward hIAPP over unrelated amyloidogenic proteins like Tau reinforces its potential for targeted therapeutic applications.

In conclusion, our findings position the JNR as a promising nanotherapeutic scaffold capable of selectively mitigating amyloidogenic toxicity in T2DM. This work lays a foundation for the rational design of anisotropic, ligand‐functionalized nanostructures tailored to intervene in protein misfolding diseases, with potential implications for early‐stage therapeutic intervention in diabetes. The functional duality possibility, inherent to the Janus architecture with two faces, was not investigated here as only one face of the JNR was functionalized. Future developments could exploit this architecture to functionalize the opposite face with cell‐targeting ligands or with a second effective drug in T2DM, paving the way for dual‐functionality precision nanomedicines.

## Author Contributions

**Mathilde Jégo**: data curation, formal analysis, investigation, methodology, validation, writing – original draft, writing – review and editing. **Sandra Kalem**: data curation, investigation, formal analysis, writing – review and editing. **Irene Antignano**: data curation, formal analysis, writing – review and editing. **David Siefker**: data curation, formal analysis, methodology, writing – review and editing. **Mingsheng Ji**: data curation, formal analysis, writing – review and editing. **Julia Kaffy**: data curation, formal analysis, writing – review and editing. **Lynda Benrabah**: data curation, formal analysis, investigation. **Chloé Caryou**: formal analysis. **Kawthar Bouchemal:** conceptualization, funding, **Mélanie Hery**: investigation, methodology. **Erwan Nicol**: investigation, formal analysis, writing – review and editing. **Sandrine Pensec**: conceptualization, investigation, methodology, supervision, writing – review and editing. **Jutta Rieger**: conceptualization, investigation, methodology, supervision, writing – review and editing. **Claire Smadja**: investigation, supervision, writing – review and editing. **Sandrine Ongeri**: conceptualization, funding acquisition, formal analysis, supervision. **Laurent Bouteiller**: conceptualization, funding acquisition, investigation, methodology, project administration, supervision, writing – review and editing. **Olivier Colombani**: conceptualization, funding acquisition, methodology, formal analysis, supervision, writing – review and editing. **Myriam Taverna**: conceptualization, funding acquisition, formal analysis, investigation, methodology, project administration, resources, supervision, writing – review and editing.

## Funding

This study was supported by the Agence Nationale de la Recherche (ANR‐21‐CE06‐0017).

## Conflicts of Interest

The authors declare no conflicts of interest.

## Supporting information

Supplementary Material

## Data Availability

The data that support the findings of this study are available on request from the corresponding authors.
