# Supplementary material for: Janus Polymeric Nanorods Inhibit human Amylin Oligomerization and Fibrillation for Potential Type 2 Diabetes Treatment
Source: Small Sci. 2026 Jul 27;6(7):e70326. doi: 10.1002/smsc.70326 (PMC13410570; doi:10.1002/smsc.70326)
Supplement: Supplementary file 1 — Supplementary Material [file SMSC-6-e70326-s001.pdf]

Supplementary Information

# Janus Polymeric Nanoparticles Inhibit hIAPP Oligomerization and Fibrillation for Potential Type 2 Diabetes Treatment

Mathilde Jégo, Sandra Kalem, Irene Antignano, David Siefker, Mingsheng Ji, Julia Kaffy, Lynda Benrabah, Chloé Caryou, Kawthar Bouchemal, Mélanie Hery, Erwan Nicol, Sandrine Pensec, Jutta Rieger, Claire Smadja, Sandrine Ongeri*, Laurent Bouteiller,* Olivier Colombani*, Myriam Taverna*

## 1. Ligand and Polymer Synthesis

### 1.1. Characterization of synthesized polymer by Size Exclusion Chromatography (SEC)

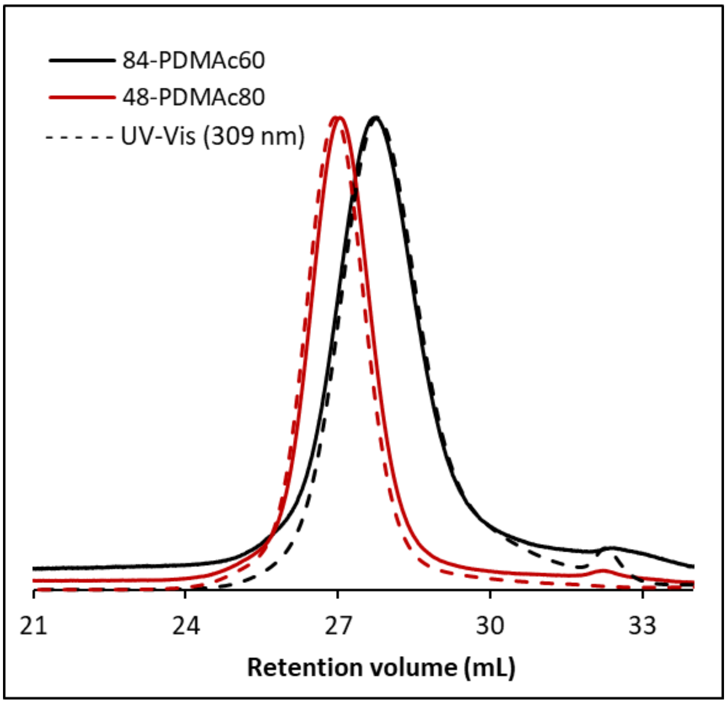


**Figure S1.** SEC trace of 84-PDMAc$_{60}$ (black) and 48-PDMAc$_{80}$ (red) in DMF (+1 g/L LiBr) with the solid lines being the normalized RI and dotted lines being the normalized UV (309 nm) signal.

## 1.2. $^{1}$H-NMR characterization of the 48-PDMAc80 polymer

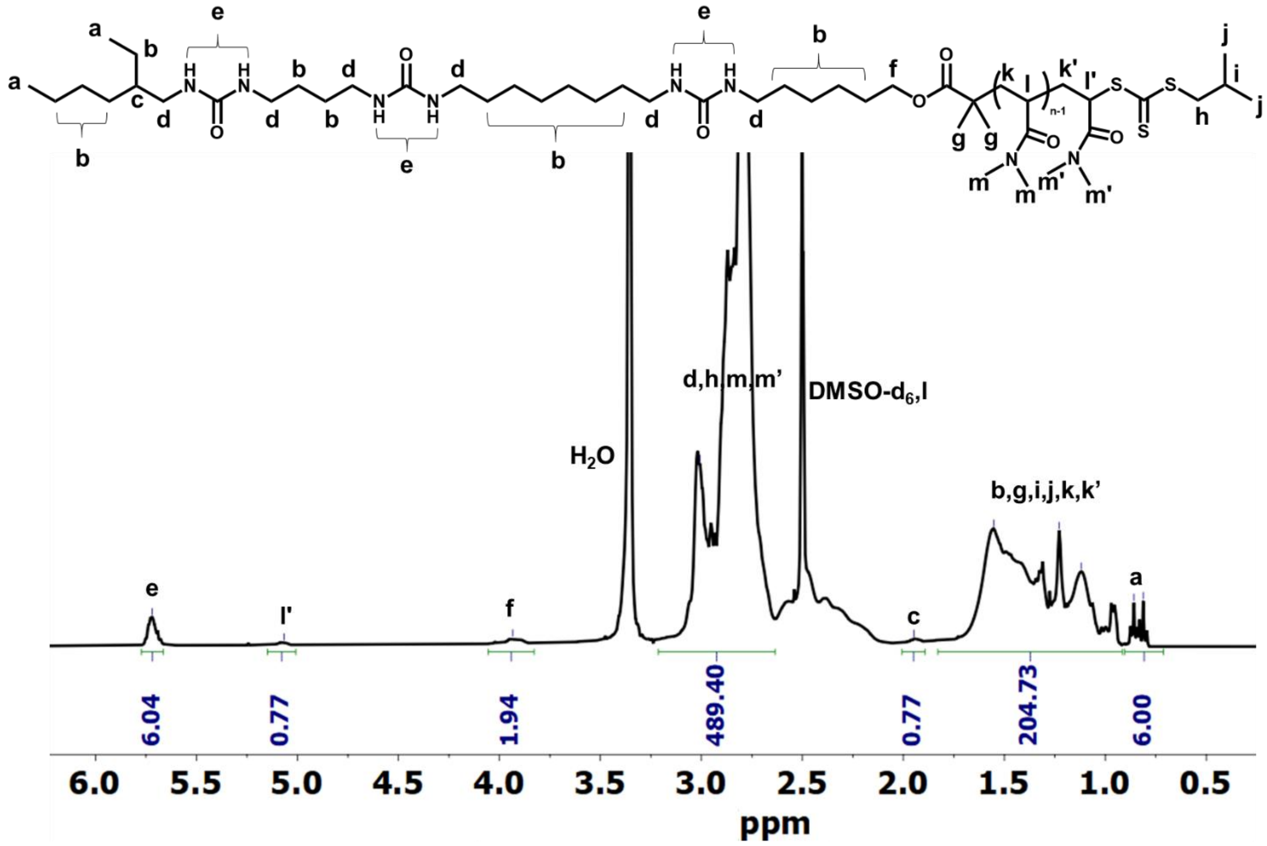


**Figure S2.** $^1$H-NMR of 48-PDMAc$_{80}$ (DMSO-d6, 400 MHz).

## 1.3. $^{1}$H-NMR characterization of the 84-PDMAc$_{60}$ polymer

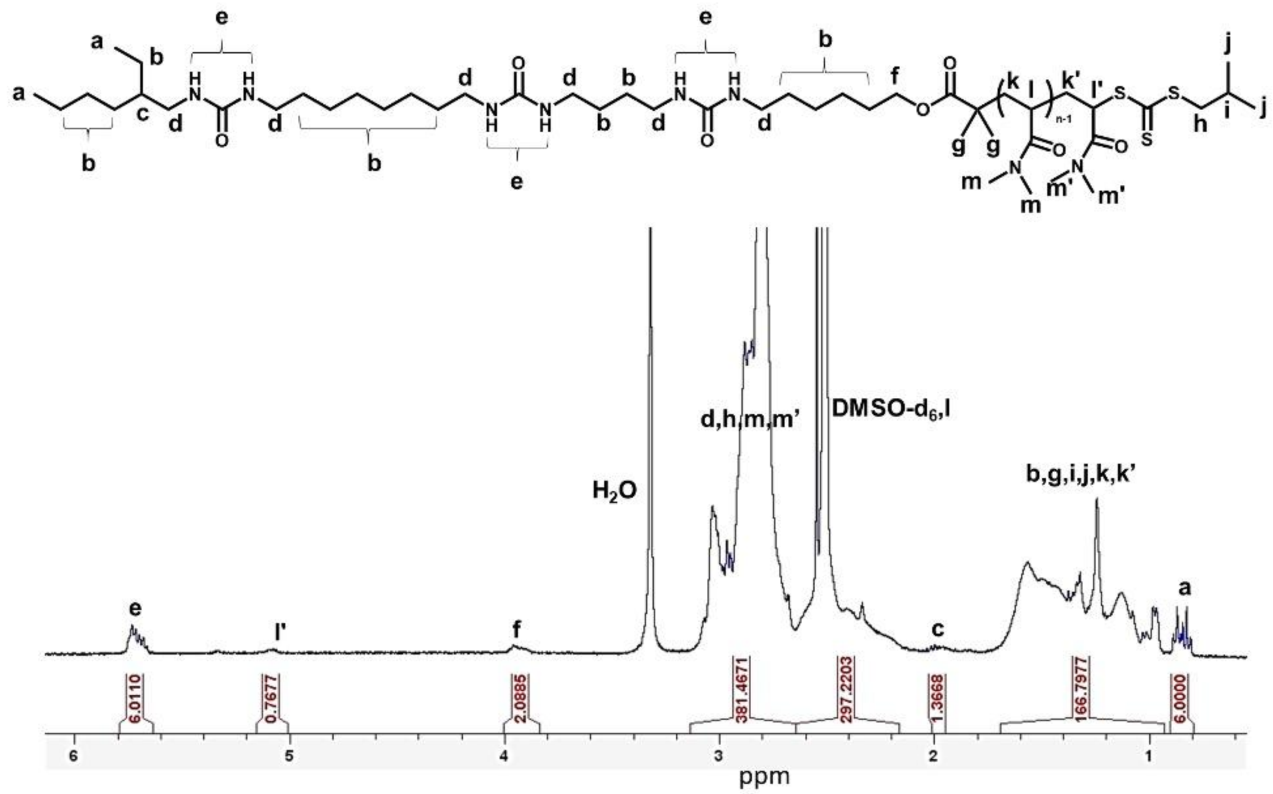


**Figure S3.** $^{1}$H-NMR of 84-PDMAc$_{60}$ (DMSO-d6, 400 MHz)**.**

## 2. UV-Vis spectrum of the polymer before and after the functionalization

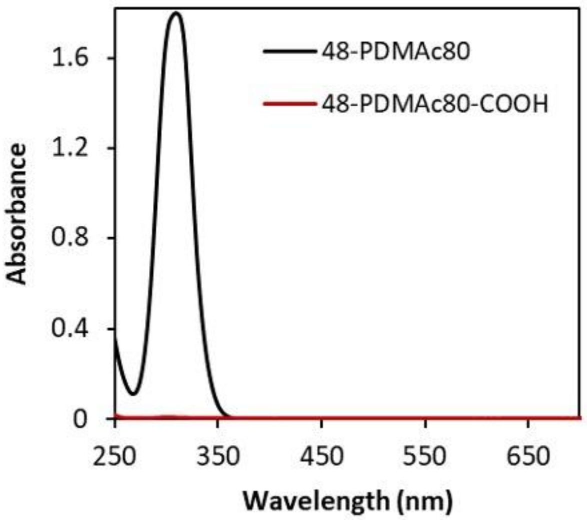


**Figure S4.** UV-Visible data of 48-PDMAc$_{80}$ (in black) and 48-PDMAc$_{80}$-COOH (in red) at 1 mg/mL in MeOH.

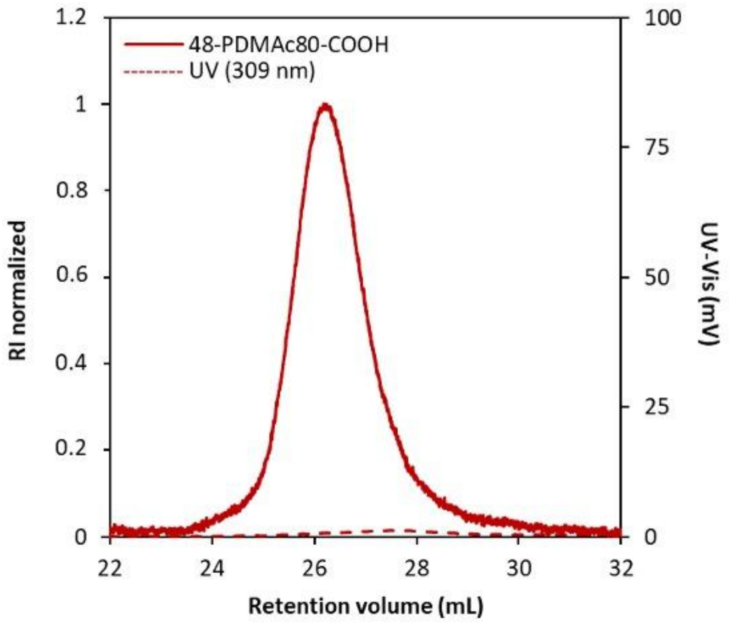


**Figure S5**. SEC traces of 48-PDMAc$_{80}$-COOH (RI signal in solid line and UV-Vis signal at 309 nm in dashed line).

## 3. Characterizations of the polymer after the functionalization

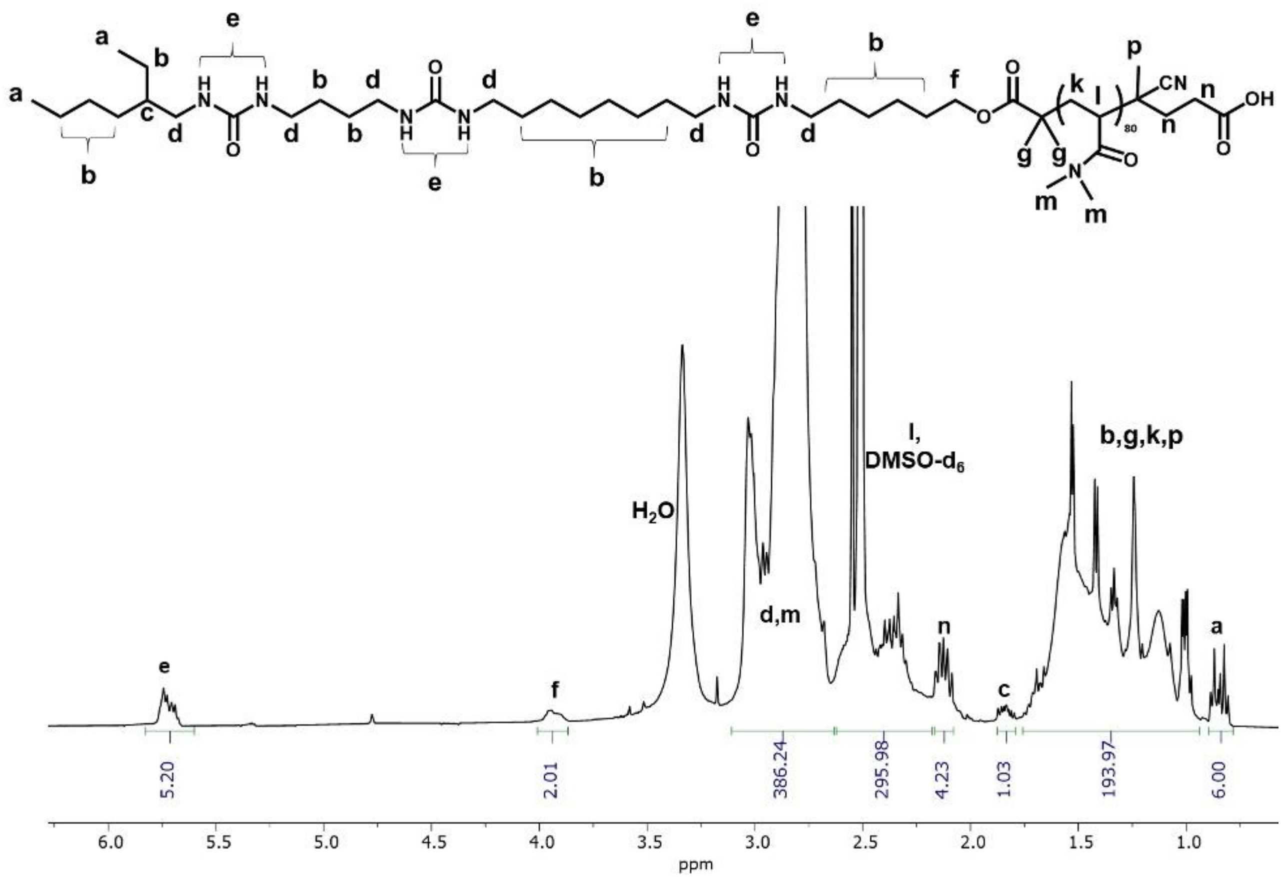


**Figure S6**. $^1$H-NMR of 48-PDMAc$_{80}$-COOH (DMSO-d6, 400 MHz).

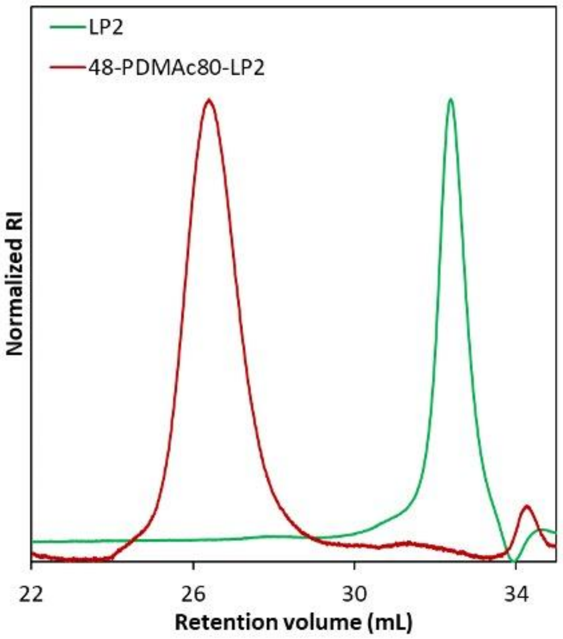


**Figure S7**. Normalized RI signals of 48-PDMAc$_{80}$-LP2 (red) and LP2 (green) obtained by SEC in DMF (+1 g/L LiBr).

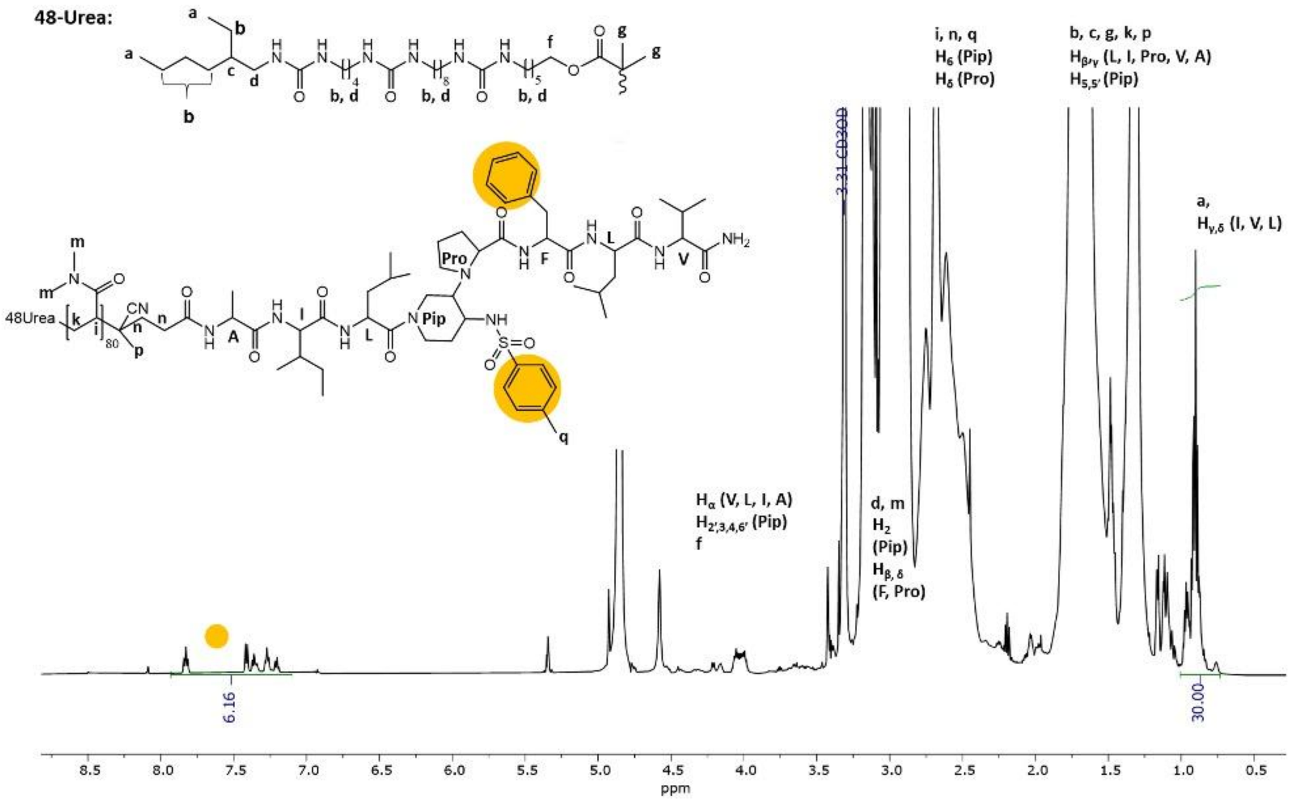


**Figure S8**. $^1$H-NMR of 48-PDMAc$_{80}$-LP2 ($CD_3OD$, 600 MHz).

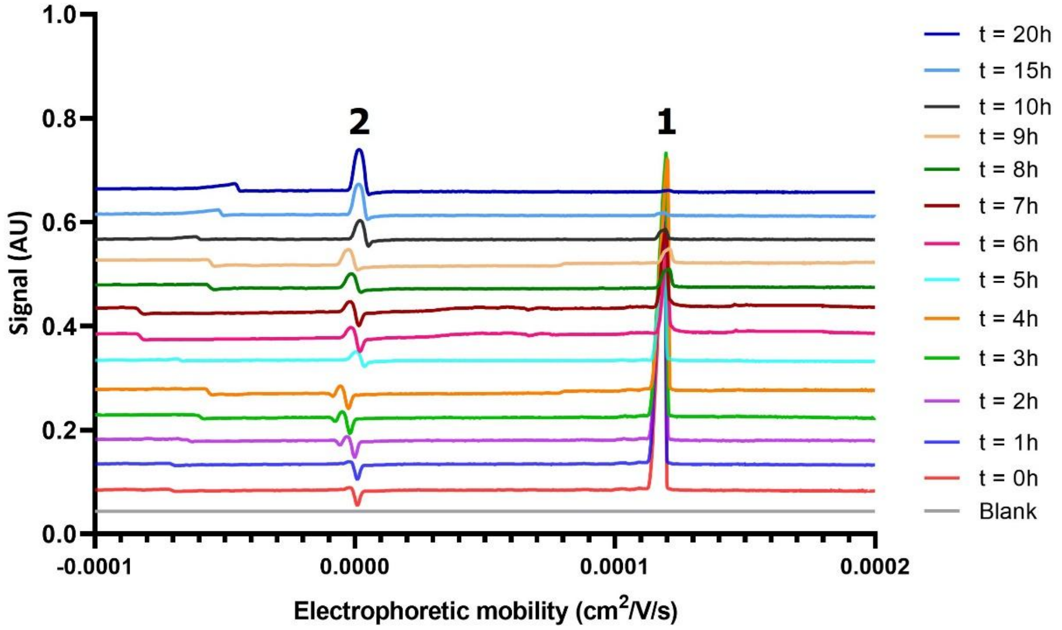


**Figure S9. Oligomerization profile of hIAPP (100 µM) over a 20-hour period**. Peak 1 corresponds to the hIAPP monomer, while Peak 2 represents oligomeric aggregates that co-migrate with the electroosmotic flow. Capillary electrophoresis conditions are identical to those described in **Error! Reference source not found.**.

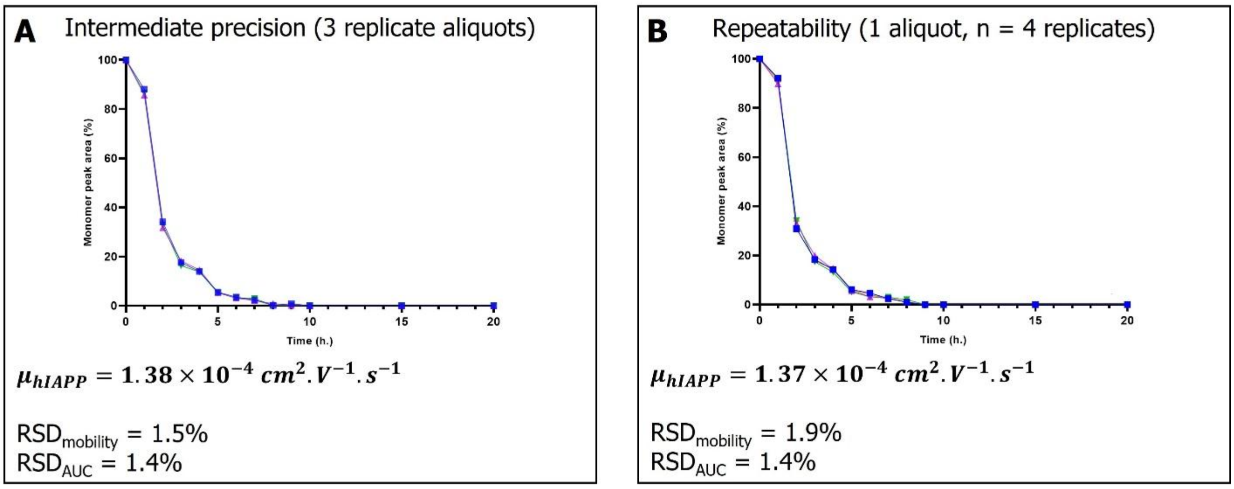


**Figure S10. Evolution of peak area percentage of hIAPP monomer upon time analyzed by CZE-UV.** (A) Assessment of intermediate precision using three distinct samples analyzed

over three separate months. (B) Evaluation of repeatability based on four consecutive measurements of a single aliquot. The electrophoretic mobility of hIAPP and the corresponding relative standard deviations (RSDs) are indicated below each graph. Experimental conditions are as described in **Error! Reference source not found.**.

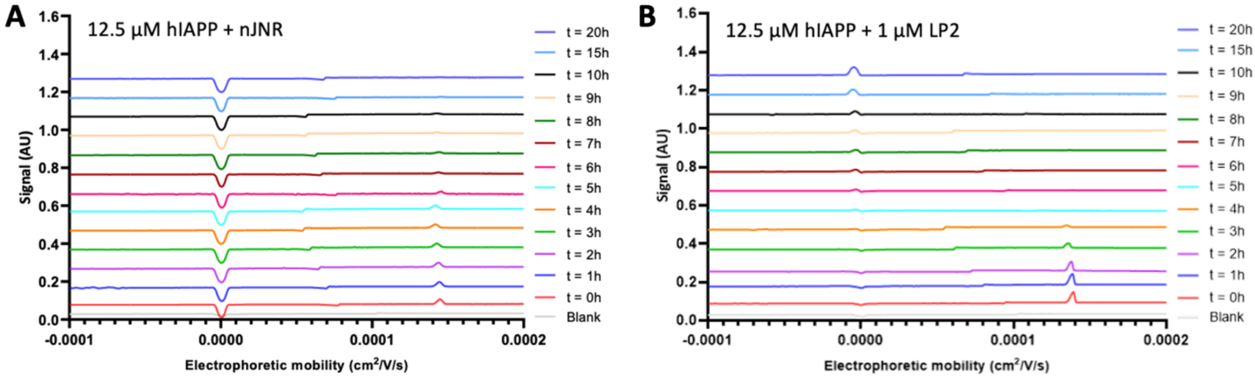


**Figure S11.** CZE profiles of 12.5 µM hIAPP incubated for 20 hours in the presence of: (A) an equivalent weight content of nJNR as JNR-LP2 in **Error! Reference source not found.**B, and (B) 1 µM free LP2. In conditions (A), the characteristic peak corresponding to higher-order hIAPP oligomers is no longer detectable, indicating a strong capacity of nJNR to sequester or disrupt soluble hIAPP aggregates. In contrast, incubation with free LP2 (B) does not result in a comparable reduction of oligomeric species. Note: The peak corresponding to free LP2, clearly visible in Figure S12 under standard conditions, is not observable here due to the elevated signal scale required for visualizing the long-term aggregation kinetics.

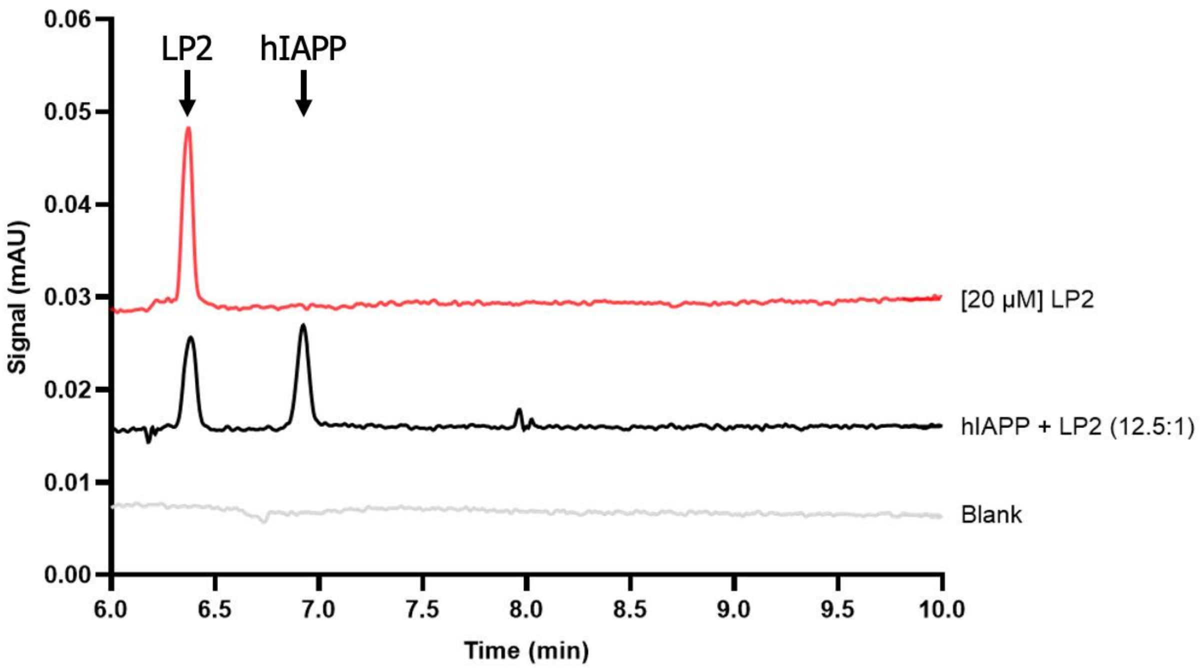


**Figure S12. Electrophoretic profile of LP2 at a concentration of 20 µM.** This electropherogram shows the migration behavior of the LP2 compound under the same experimental conditions used for hIAPP aggregation studies (as described in **Error! Reference source not found.**). A single, well-defined peak is observed, confirming the electrophoretic homogeneity of LP2 and the absence of self-association or aggregation under these conditions. Notably, the migration time of LP2 does not overlap with that of hIAPP monomers or oligomers, allowing clear spectral discrimination in co-incubation experiments.

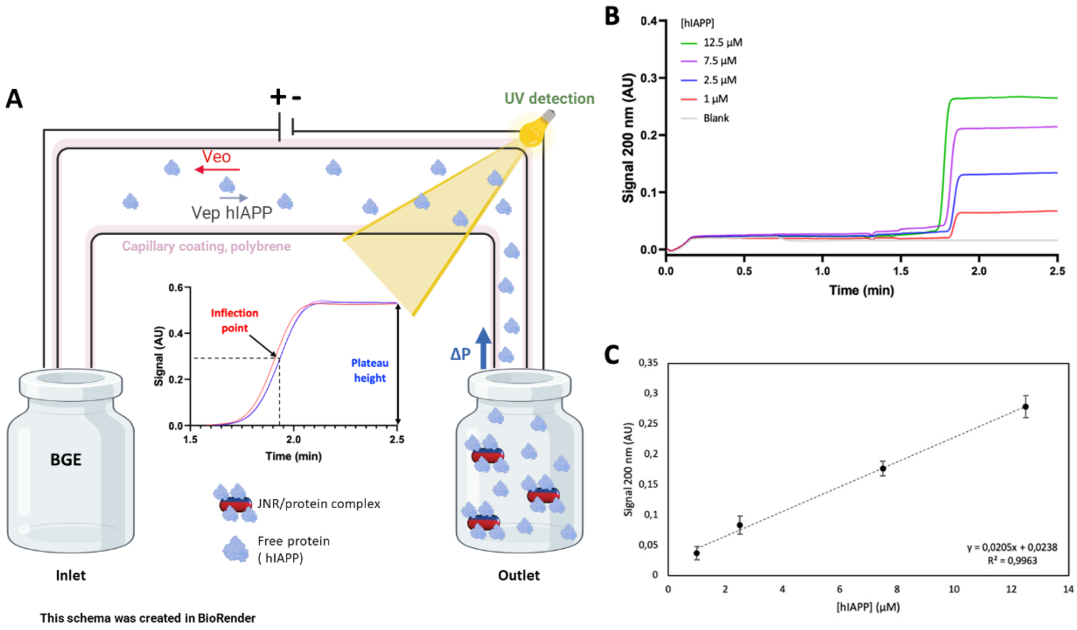


**Figure S13**. FACCE experiment of hIAPP used as the calibration for the subsequent quantification of the hIAPP-JNR interaction. (A) Schematic representation of the FACCE set-up. $V_{eo}$ represents the velocity of the electroosmotic flow, $V_{ep}$ is the velocity due to the effective electrophoretic mobility of free hIAPP. (B) Representative electropherograms of hIAPP at concentrations ranging from 1 to 12.5 µM, obtained using the FACCE method. Each signal is the mean of three independent experiments. (C) Corresponding calibration curve with error bars representing the standard deviation from the three independent experiments. Experimental conditions: hIAPP was dissolved in 50 mM ammonium acetate buffer (pH 3.7) and electrokinetically injected from the short end of a fused silica capillary (effective length: 10 cm; total length: 60 cm), pre-coated with 0.2% polybrene. Separation was carried out under an applied voltage of 20 kV with a co-injection pressure of 30 mbar.

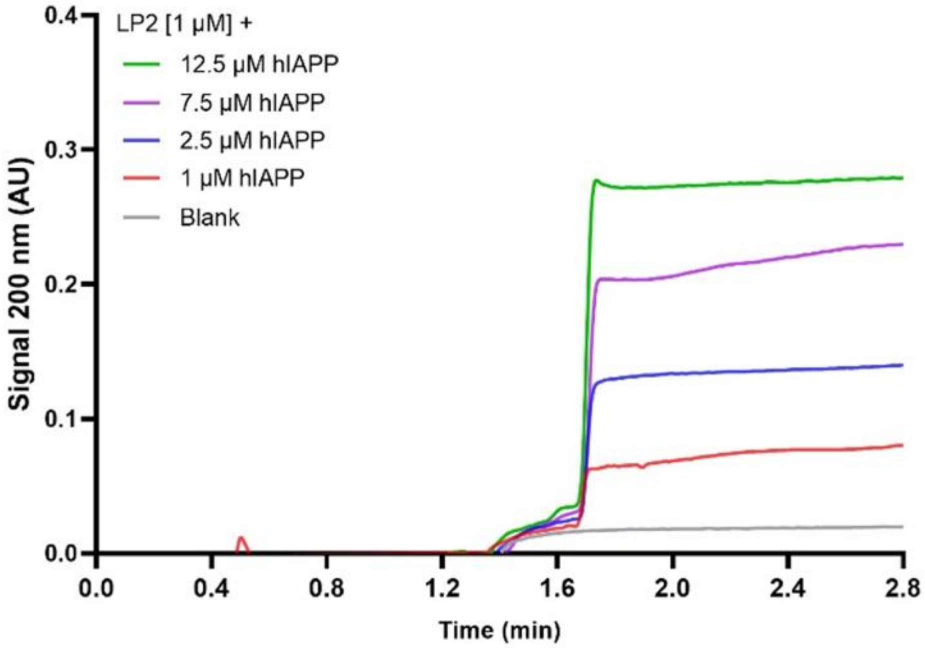


**Figure S14**. FACCE electropherograms of free hIAPP following incubation with LP2 at a fixed concentration (1 μM) and varying hIAPP concentrations. Each electropherogram represents the average of three independent experiments, with a standard deviation of < 0.006 for the plateau height values.

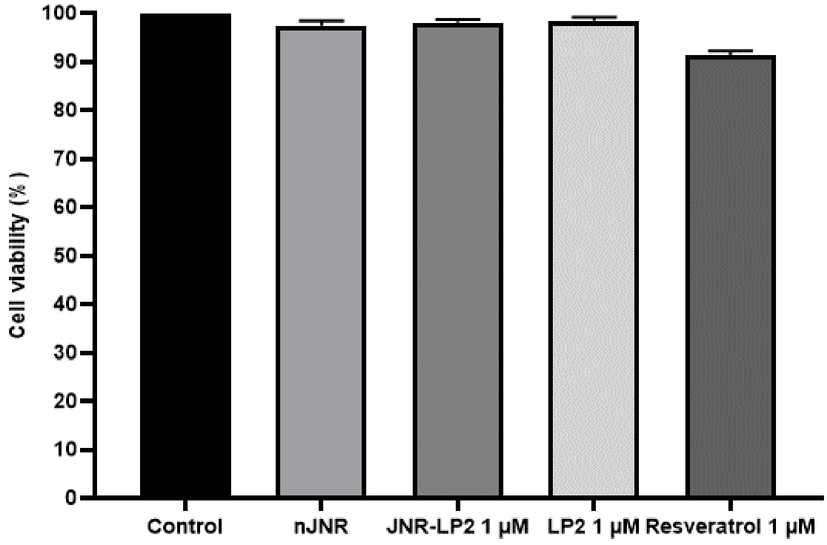


**Figure S15**. Biocompatibility assessment of the nJNR, JNR-LP2, LP2, and resveratrol. INS-1 cell viability was evaluated by MTT assay after 24 hours of exposure to RPMI medium

(control) or individual compounds (JNR-LP2, LP2, and resveratrol at a concentration of 1 µM, and nJNR as equivalent JNR-LP2 volume). None of the tested compounds exhibited detectable cytotoxicity under these conditions, with viability comparable to untreated controls. Data are presented as mean ± SD from three independent experiments.

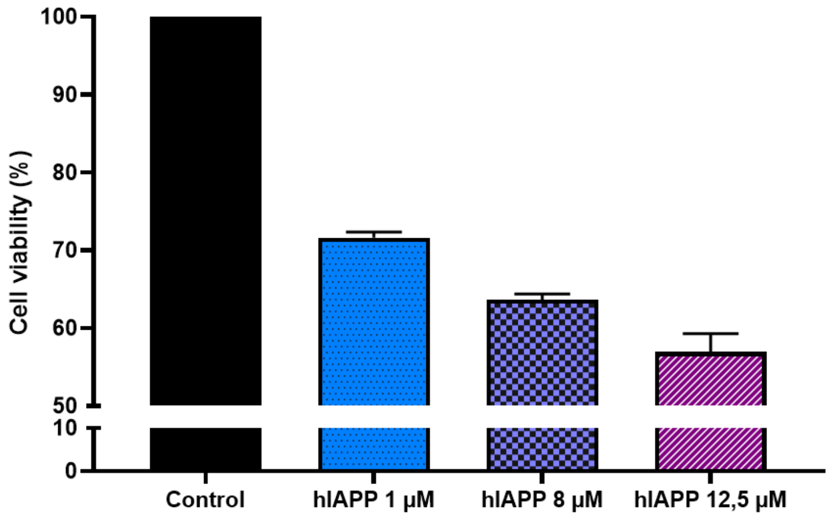


**Figure S16**. **Dose-dependent cytotoxicity of hIAPP on INS-1 pancreatic β-cells.** Cells were treated with increasing concentrations of hIAPP (1, 8, and 12.5 µM) for 24 hours, and viability was assessed using the MTT assay. A significant, concentration-dependent reduction in cell viability was observed, consistent with the well-established toxic effects of hIAPP oligomers. Data are shown as mean ± SD from three independent experiments.

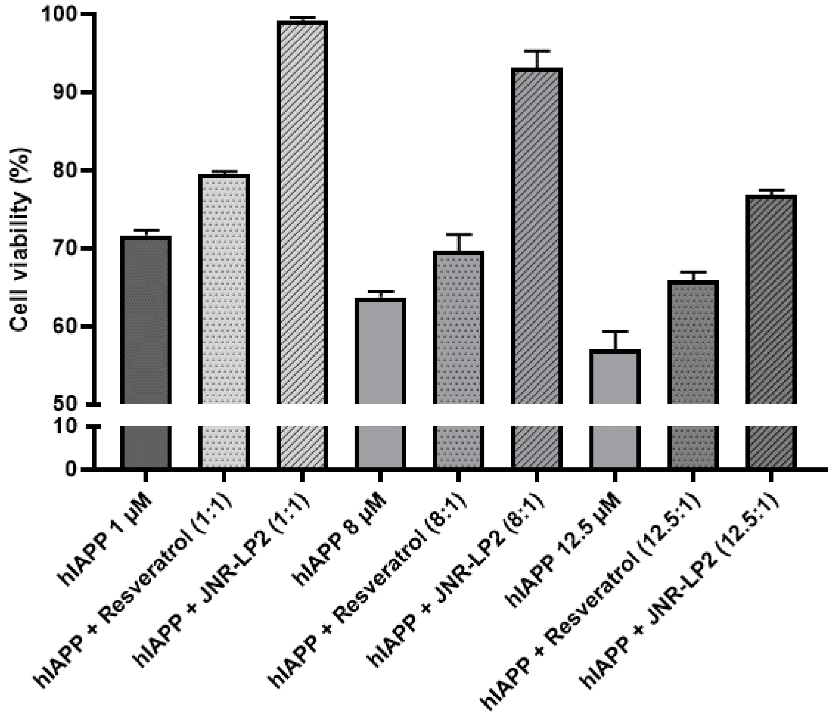


**Figure S17**. **Comparative protective efficacy of JNR-LP2 and resveratrol against hIAPP-induced cytotoxicity.** INS-1 cells were co-incubated with hIAPP (1, 8, or 12.5 µM) and either resveratrol (1 µM) or JNR-LP2 (1 µM) at the indicated molar ratios for 24 hours. Cell viability was assessed via the MTT assay. JNR-LP2 exhibited superior cytoprotective effects compared to resveratrol across all tested conditions. Data are presented as mean ± SD from three independent experiments.
